# Impact of searching clinical trials registers in systematic reviews of pharmaceutical and non‐pharmaceutical interventions: Reanalysis of meta‐analyses

**DOI:** 10.1002/jrsm.1583

**Published:** 2022-07-28

**Authors:** Zainab Alqaidoom, Phi‐Yen Nguyen, Maryam Awadh, Matthew J. Page

**Affiliations:** ^1^ School of Public Health and Preventive Medicine Monash University Melbourne Australia; ^2^ School of Medicine Southeast University Nanjing China

**Keywords:** meta‐analysis, publication bias, systematic review, trial registration

## Abstract

Systematic reviewers are advised to search trials registers to minimise risk of reporting biases. However, there has been little research on the impact of searching trials registers on the results of meta‐analyses. We aimed to evaluate the impact of searching clinical trials registers for systematic reviews of pharmaceutical or non‐pharmaceutical interventions. We searched PubMed, Scopus, Science Citation Index and Social Sciences Citation Index, and Education Collection for systematic reviews with meta‐analyses indexed from 2 November to 2 December 2020. A random sample of systematic reviews was initially drawn, and for reviews which considered randomised trials eligible for inclusion, which had not searched a trials register, we searched ClinicalTrials.gov, EudraCT, ANZCTR, and the WHO ICTRP search portal for eligible trials. We compared meta‐analytic effect estimates before and after including results from additional trials identified. We found additional trials for 63% (63/101) of eligible reviews; however, trials with results that could contribute to a meta‐analysis were identified for only 20% (20/101) of the reviews. On average, there was no difference in the meta‐analytic effect estimates before versus after adding the new trials. In summary, searching clinical trial registers led to identification of additional trials for many reviews; however, very few trials had results available for inclusion in meta‐analyses. Including results from the new trials led to no change in the meta‐analytic estimates, on average. Trials registers would be even more valuable to systematic reviewers if more trialists made use of them (i.e., registered their trials and posted results in a timely manner).

**Highlights**

- There has been limited investigation into how often searching clinical trials registers (e.g., ClinicalTrials.gov) yields additional trials not otherwise identified, and what impact including results from registered trials has on the results of meta‐analyses.
- In a sample of 101 systematic reviews which had not searched trials registers, searching ClinicalTrials.gov, EudraCT, ANZCTR and the WHO ICTRP search portal identified at least one new eligible trial for 63% of reviews. For 20 reviews, the new trial(s) identified had results available for inclusion in a meta‐analysis. However, there was no difference in the meta‐analytic risk ratios (combined ratio of risk ratios 1.00, 95% confidence interval 0.99–1.01) and the meta‐analytic standardised mean differences (combined difference in standardised mean differences 0.01, 95% confidence interval −0.04 to 0.06) before versus after adding the new trials.
- Searching clinical trials registers can help identify additional trials to be included in a systematic review. Such trials might include results available for inclusion in meta‐analyses and can inform assessments of the risk of reporting bias. Fields which are seeing increasing rates of pre‐registration of studies (e.g., psychology) should consider making use of study registers when conducting systematic reviews.

## 1 BACKGROUND

Publication bias and selective non‐reporting bias are two major threats to the validity of systematic reviews and meta‐analyses of randomised controlled trials.^1^ Publication bias occurs when journals tend to accept trials based on the statistical significance, magnitude or direction of effect estimates.^2^ Trials with large or positive treatment effects tend to be published more often and quicker than trials with smaller treatment effects or negative results.^3^ Selective non‐reporting bias (also known as outcome reporting bias) occurs when authors selectively omit results of some outcomes (particularly those that are unfavourable to the experimental intervention), despite those outcomes having been measured.^4^ Mandatory trial registration has been proposed as a mechanism to reduce publication bias and selective non‐reporting bias; it is assumed that by publicly declaring the existence of a trial, trialists will be motivated to fully publish their trial and report results for all outcomes, and any deviation from the trial registration record will be noticed.^5^ Searching trials registers is therefore recommended as a useful strategy to identify ongoing and completed trials, regardless of whether they are published or not, and hence allows systematic reviewers to explore the risk of reporting bias.^6^

Previous studies have indicated underutilization of searching trials registers in most medical systematic reviews.^7^, ^8^, ^9^, ^10^, ^11^, ^12^, ^13^, ^14^, ^15^, ^16^ In a cross‐sectional study of 117 systematic reviews that were published between 1 July 2012, and 30 June 2013, in six high impact medical journals, only 41 (35%) reviews reported searching a trials register.^15^ This study and others have found the percentage of reviews searching trials registers varied from one medical specialty to another. Trials register searching has been reported as 6% in neurology reviews, 7% in orthopaedics surgery reviews, 11% in dermatology reviews, 12% in anaesthesiology reviews, 17% in general urology reviews, 18% in obstetrics and gynaecology reviews, 13%–25% in surgery reviews, and 20% in emergency medicine reviews.^7^, ^8^, ^9^, ^10^, ^11^, ^12^, ^13^, ^14^, ^16^ On the other hand, Berber et al. conducted a cross‐sectional study of systematic reviews from all Cochrane groups published through 1 February 2017 and found that over 90% of Cochrane authors search a trials register.^17^

Only a few studies have evaluated how often additional trials are identified via searching trials registers, and the impact of including the new trials on meta‐analysis results. Fuller et al. examined a random sample of 25 minimally invasive surgical oncology reviews and found new trials for 64% (16/25) of the reviews.^12^ Similarly, Reddy et al. retrieved additional trials from trials registers for 59% (59/100) of orthopaedic surgery reviews.^14^ To our knowledge, Baudard et al. conducted the first investigation of the impact of searching trials registers on meta‐analysis results.^18^ A random sample of systematic reviews of pharmaceutical interventions published between June 2014 to January 2015 were included, regardless of the specialty.^18^ They found additional trials for 43% (41/95) of the reviews, and the increase in the number of patients included ranged from 10% to 50%.^18^ Results were identified for 52% (63/122) of the additional trials located, involving 42,202 participants, in which 71% (45/63) of trials involving 21,358 participants were included in the re‐analysis for 14 reviews.^18^ Including results of any additional trials found in ClinicalTrials.gov in the original meta‐analyses resulted in an increase in precision and change in the summary effect estimate in several reviews; however, none of the changes led to a change in interpretation of the results.^18^ In addition, Wilson et al. investigated if searching ClinicalTrials.gov would change the conclusion of a systematic review of interventions for peripheral diabetic neuropathy; 106 trials were identified, 23 in ClinicalTrials.gov; however, including those 23 trials did not change the conclusion or statistical significance of the results for the two outcomes investigated.^19^

Baudard et al. limited their investigation to a search of the World Health Organisation International Clinical Trials Registry Platform (WHO ICTRP) only and the impact of searching it on systematic reviews of pharmaceutical interventions published in 2014–2015, and Wilson et al. investigated the impact of searching clinical trials in one systematic review.^18^, ^19^ To address these limitations, we undertook this methodological study to assess the impact of searching various clinical trial registers on results of systematic reviews of pharmaceutical and non‐pharmaceutical interventions published in 2020.

## 2 METHODS

### 2.1 Identification of the systematic reviews

This study arose out of the REPRISE (REProducibility and Replicability in Syntheses of Evidence) project.^20^ The REPRISE project consists of four studies that aim to investigate the transparency, reproducibility and replicability of systematic reviews of interventions. Full details about the methods for all REPRISE studies can be found in the REPRISE protocol,^20^ and the results will be reported elsewhere. Here, we report the methods used in our study exploring the impact of searching trials registers on the results of systematic reviews; a protocol for these methods was not published.

In the current study, systematic reviews meeting the following criteria were considered eligible for inclusion:

1. Written in English.
2. The review objectives or questions were clearly reported in the introduction or methods.
3. The search sources (e.g., bibliographic databases) consulted were reported.
4. The validity assessment tools, such as risk of bias or methodological quality assessment tools were stated in the methods or results section.
5. The review included studies comparing the effects of at least two health, behavioural, social, or educational interventions.
6. The full bibliographic citations of included studies must have been presented.
7. Review authors considered either randomised trials only, or randomised and non‐randomised studies, as eligible for inclusion.
8. Review authors did not perform any clinical trials register search.
9. The search strategy (including terms for population and interventions), and dates of database searches, were reported. An exception was made for reviews in which the population was healthy adults, and so the search terms for intervention and outcomes needed to have been reported. Moreover, reviews with complex search strategies (i.e., where it was difficult to determine which were the population, intervention, comparison or outcome components) were excluded.

An information specialist created the search strategies to identify eligible systematic reviews (see Data S1). We searched the following databases for systematic reviews indexed between 2 November 2020 and 2 December 2020: PubMed, Scopus (via Elsevier), Science Citation Index and Social Sciences Citation Index (via Web of Science), and Education Collection (via ProQuest). After removing duplicate records using Endnote software, the unique records yielded from the search were randomly ordered and the first 2000 records were imported into Covidence for screening. Two authors independently screened the titles/abstracts of these 2000 records. Full‐texts of potentially eligible citations were retrieved and evaluated for eligibility independently by two authors (again in a randomly sorted order) until a total of 302 systematic reviews meeting inclusion criteria 1–6 were included. Disagreements were resolved by discussion between authors.

### 2.2 Data extraction from the systematic reviews

For each systematic review meeting the inclusion criteria 1–6 of the REPRISE project, two authors independently extracted data on general characteristics of the review and reporting characteristics using a standardised form created in REDCap v10.6.12. General characteristics included author name and country, type of interventions and health conditions (ICD‐11), number of included studies, journal name, funding source, conflict of interest, and protocol or registration status of the review. Investigators also recorded which study designs were reported as eligible for inclusion, and whether the systematic reviewers reported conducting a search of a trials register. Other reporting characteristics captured are summarised elsewhere, as they are beyond the scope of the current study. Disagreements in data extracted were resolved by discussion between investigators. For those reviews meeting inclusion criteria 1–9, the following additional items were extracted by one author (Z. A.): inclusion and exclusion criteria for the review, search terms related to the population and intervention (or intervention and outcome, as applicable), and dates in which searches of databases were run.

### 2.3 Searching clinical trials registers

For all systematic reviews meeting inclusion criteria 1–9, one author searched the following trials registers and search portals: ClinicalTrials.gov, the European Union Drug Regulating Authorities Clinical Trials Database (EudraCT), the Australian New Zealand Clinical Trials Registry (ANZCTR), and the World Health Organisation International Clinical Trials Registry Platform (WHO ICTRP) search portal. We chose to search Clinitaltrials.gov, EudraCT, and ANZCTR registers, because they allow trialists to post results directly in the register, and we searched the WHO ICTRP search portal because it collects multiple registers and Cochrane recommends review authors search it. We used in our search strategies the search terms relating to the population and intervention (or intervention and outcome, where applicable), as sought from the systematic reviews. Data S2 provides details on the steps we followed to search each register. Data S3 provides details of the search strategies we used for each systematic review.

We had to adjust search strategies individually for each register. Each trials register has different word/character limits, for example, there is a 250‐character limit for the condition or disease and other terms search boxes in ClinicalTrials.gov, and a 100‐character limit for the ANZCTR basic search toolbar. There were no search word limits in the basic search toolbars for EudraCT and the WHO ICTRP search portal; however, we noticed that using a very long search strategy compromised the search and resulted in identifying zero records or error messages. Moreover, basic search functions were used for ANZCTR and WHO, as there were similar defaults if using advanced search functions.

For very long search strategies, we had to run the search multiple times to make sure we included all possible terms, for example, if 20 different terms were provided for intervention, and the register only accepted fewer than 10 terms, we did the following: entered (Population terms) AND (Intervention terms 1–10) and ran the search, then entered (Population terms) AND (intervention terms 11–20) and ran the search, and after that, we combined the records and eliminated the duplicates.

After running each search, we exported all records into Microsoft Excel. The records identified via the WHO ICTRP search portal which came from ClinicalTrials.gov, EudraCT, and ANZCTR were marked by us as ‘no need to screen’, given we had already run searches of these three registers. We set ‘500 records per register’ as our screening threshold for each systematic review; that is, whenever a search of a register yielded more than 500 records to screen, after having already removed records completed after the date of the last search of the systematic review, that systematic review was excluded from our study as we had limited time and resources to screen all records. After applying this threshold, one author (Z. A.) screened each trials register record against the eligibility criteria stated by the original systematic reviewers, marking whether the trial had any results posted in the trials register or not. We screened each full registration record at the source register website (i.e., not just the information exported into Excel). Furthermore, for trials that met the reviews' eligibility criteria and reported any of the outcomes of interest to the review, but which had no results posted in the register or no publication details provided in the register, we undertook a Google search, using the trial title and cross‐checking other details (e.g., the authors' names, sponsors, and abstract) to confirm the match, and retrieved the publications if applicable.

All trials registration records yielded by the searches for a random sample of 20 reviews were screened independently by two authors (Z. A. and either M. A., P. N. or M. J. P.). We calculated the percentage agreement between the two authors in trials being recorded as meeting the eligibility criteria of the review and the percentage of reviews for which the first author needed to change their screening decisions after discussing discrepancies with the second author.

We classified systematic reviews that had at least one potentially eligible trial meeting the review eligibility criteria into the following three categories:

1. Had at least one new trial meeting the review eligibility criteria, without any trial results available.
2. Had at least one new trial meeting the review eligibility criteria, with trial results available, but not necessarily for inclusion in any of the meta‐analyses;
3. Had at least one new trial meeting the review eligibility criteria, with trial results available for inclusion in one of the meta‐analyses.

### 2.4 Collection of outcome data for the additional trials

The outcome domains (e.g., pain, hepatocellular carcinoma incidence, quality of life) of all meta‐analyses reported in each systematic review were noted and compared with the outcome domains of all results available for the new trials identified (e.g., posted in the register or published in a journal), to see whether the trials could contribute to any of the meta‐analyses. Results needed to be made available in the register (or in a publication) before the date in which the original systematic reviewers last conducted their searches for studies. If the new trial(s) could contribute data to more than one meta‐analysis in a review, and we considered the trial(s) sufficiently similar to the already included trials (hence it made clinical sense to incorporate the new trials in the meta‐analysis), we extracted data for the outcome that was measured most frequently across the new trials. For example, if data were available for pain in three new trials and for quality of life in one new trial, we extracted data from the three trials with pain data only, for inclusion in the meta‐analysis of pain. This procedure was followed so that only one meta‐analysis per systematic review was included in our analysis.

One investigator (Z. A.) extracted data from the new trials with results available into Microsoft Excel sheets. Data extracted from the new trials for binary outcomes included sample size and the number of events in each group. For continuous outcomes, sample size, means and standards deviations for each group were extracted from the new trials. For the original studies already included in the systematic reviews, similar data were extracted if reported on the forest plot, and if not, we extracted effect estimates (e.g., risk ratio, mean difference) and its 95% confidence intervals. Moreover, all outcome data extracted from trial registers or publications, and from forest plots, were double‐checked and verified for accuracy by a second investigator (M. J. P.).

### 2.5 Data analysis

All statistical analyses were conducted using Stata (17.0, StataCorp LLC, College Station, TX). We calculated summary statistics to describe the characteristics of included systematic reviews (frequencies and percentages for categorical variables, median and interquartile ranges [IQR] for continuous variables). We reported frequencies and percentages, and calculated medians, IQRs, and ranges of the numbers of records yielded from the searches of each trials register, and the numbers of records that met the review inclusion criteria and measured the outcomes of interest to the systematic reviewers.

We reanalysed the original meta‐analyses by including the outcome data from the new trials. We used similar effect measures (e.g., mean difference) and statistical models and methods (e.g., random‐effects model, DerSimonian–Laird method) used by the original authors, except for one case of a meta‐analysis of hazard ratios, where we reanalysed the data using the risk ratio because new trials did not report hazard ratios.

We calculated the differences between the meta‐analytic effect estimates before and after adding the new trials. For meta‐analyses of binary outcomes, we analysed study data as risk ratios (RRs) and used the ratio of risk ratios (RRRs) to estimate the difference in meta‐analytic estimates. For meta‐analyses of continuous outcomes, we analysed study data as standardised mean differences (SMDs) and used the difference in standardised mean differences (dSMDs) to estimate the difference in meta‐analytic estimates. The direction of effect for meta‐analytic estimates was standardised so that RRR and dSMD estimates below the null indicated that the meta‐analytic estimate was more favourable to the experimental intervention before, rather than after, adding the new trials. We synthesised the RRR and dSMD estimates using a random‐effects meta‐analysis model, with the meta‐analytic weights based on the variance of the original meta‐analytic estimate and the between study variability estimated using the restricted maximum likelihood estimator. The Hartung–Knapp–Sidik–Jonkman confidence interval method was used to calculate uncertainty in the combined differences.^21^, ^22^ We quantified statistical inconsistency using the *I* ^2^ statistic^23^ and generated a 95% predictive interval for the combined RRR and dSMD, respectively.

## 3 RESULTS

**FIGURE 1:**
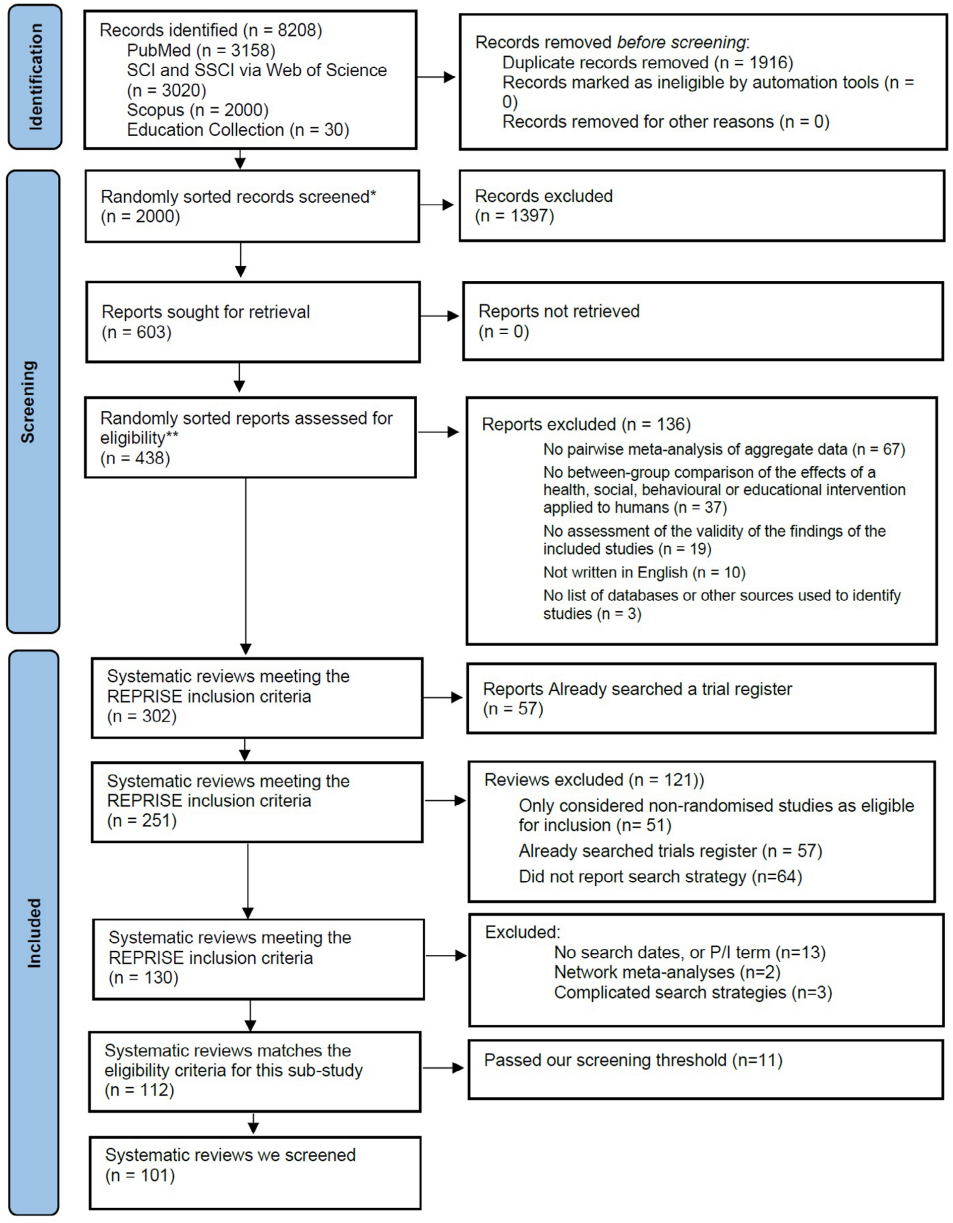
PRISMA 2020 flow diagram of identification, screening and inclusion of systematic reviews. *There were 6292 unique records after duplicates were removed, but we only needed to screen 2000 randomly sorted records to reach our target sample size of 302 reviews. **We only needed to screen 438 of the 603 full text reports retrieved to reach our target sample size of 302 reviews [Colour figure can be viewed at wileyonlinelibrary.com]

We identified 302 systematic reviews meeting inclusion criteria 1–6 (Figure 1). After removing 51 reviews which only considered non‐randomised studies as eligible for inclusion, we found that in 57/251 (23%) of the remaining reviews, at least one trials register was searched. After removing these 57 reviews and 64 reviews which did not report a search strategy for at least one bibliographic database, we were left with 130 reviews. We excluded 17 of these reviews for the following reasons: 13/130 did not report the dates of searches, 2/130 were network meta‐analyses (for which we do not have the data necessary to reanalyse), and 3/130 had complicated search strategies. We ran searches of trials registers for the remaining 112 reviews which met inclusion criteria 1–9, however, 11 passed our ‘500 records per register’ screening threshold and hence the registration records were not screened. Thus, we screened trials register records for 101 reviews.

### 3.1 Basic characteristics of included systematic reviews

Table 1 indicates the characteristics of 101 systematic reviews for which we screened trials registration records. The majority of the systematic reviews included addressed multidisciplinary health interventions (e.g., nutrition, physiotherapy, psychology) (33% [33/101]), followed by medical pharmaceutical interventions (29% [29/101]), surgical interventions (21% [21/101]), traditional Chinese medicine interventions (9% [9/101]), and dental interventions (5% [5/101]). According to the ICD‐11 health condition classification, the majority of health conditions addressed in the included reviews were diseases of the digestive system (15% [15/101]), endocrine, nutritional or metabolic diseases (14% [14/101]), diseases of the musculoskeletal system (13% [13/93]), and neoplasms (9% [9/101]). The median number of included studies in the reviews was 12 (IQR 8–18; range 3–64).

**TABLE 1:** Characteristics of the 101 included systematic reviews for which we screened trials registration records

Characteristic	Frequency (%)
Country of corresponding author	
China	30 (30)
United States of America	10 (10)
Brazil	7 (7)
Spain	7 (7)
Taiwan	6 (6)
United Kingdom	6 (6)
Others	35 (35)
Type of health condition addressed (ICD‐11)	
Diseases of the digestive system	15 (15)
Endocrine, nutritional or metabolic diseases	14 (14)
Diseases of the musculoskeletal system	13 (13)
Neoplasms	9 (9)
Certain infectious or parasitic disease	6 (6)
Diseases of the nervous system	6 (6)
Other health condition	38 (38)
Number of included studies^a^	12 (8–18)
Subgroup of interventions addressed	
Medical—Pharmaceutical interventions	29 (29)
2. Surgical interventions	21 (21)
3. Medical—Both medical and surgical interventions^b^	4 (4)
4. Dental interventions	5 (5)
5. Multidisciplinary health—Nutritional interventions	14 (14)
6. Multidisciplinary health—Physiotherapy interventions	7 (7)
7. Multidisciplinary health—Psychology interventions	12 (12)
8. Traditional Chinese medicine—Herbal and non‐herbal interventions	9 (9)
Source of funding	
Non‐profit	43 (43)
For‐profit	1 (1)
Both non‐profit and for‐profit	2 (2)
Authors specified there was no funding	32 (32)
Not reported	23 (23)
Financial conflicts of interest of systematic reviewers	
At least one systematic reviewer reported a financial conflict of interest	10 (10)
All systematic reviewers stated they had no financial conflicts of interest	89 (88)
No disclosure statement	2 (2)
Systematic review protocol or registration mentioned	
Both a protocol and registration record cited	1 (1)
Only a protocol cited	2 (2)
Only a registration record cited	46 (46)
Neither cited	52 (52)

^a^ Data are given as median (interquartile range).

^b^ Were reclassified in both the medical and surgical categories for the analysis.

Most corresponding authors had affiliations from China (30% [30/101]) and the United States of America (10% [10/101]). Authors stated that no funding was received in 34% (32/93) of reviews, and funding was received from non‐profit sources in 43% (43/101) of reviews. In 89% (83/101) of reviews, all review authors stated they had no financial conflict of interest. In 52% (52/101) of reviews, authors did not report working from a protocol or registration record.

### 3.2 Results of registers search

After removing records marked as 'no need to screen', records of trials completed after the search dates for the included systematic reviews and eliminating ongoing trials from EudraCT, ANZCTR, and WHO ICTRP registers (we did not need to do this for ClinicalTrials.gov records as we placed a date limit in the register search), we retrieved a total of 34,391 records for the 112 reviews for which we ran trials register searches. Considering each register separately, 23,598 were from ClinicalTrials.gov, 1884 from EudraCT, 1784 from ANZCTR, and 7131 from the WHO ICTRP search portal. The median number of records yielded per review was 39 (IQR 8–114 [range 0–12,063]) from all registers, and according to each register: 17 (4–63 [0–9768]) from ClinicalTrials.gov, 0 (0–3 [0–596]) from EudraCT, 0 (0–4 [0–401]) from ANZCTR, and 3 (0–14 [0–1894]) from the WHO ICTRP search portal (Table 2).

**TABLE 2:** Overall median (IQR [range]) for the total number of trials yielded by the searches per review, and by each register (*n* = 112)

All registers	ClinicalTrials.gov	EudraCT	ANZCTR	WHO ICTRP
Overall numbers for all systematic reviews (*n* = 112)				
39 (8–114 [0–12,063])	17 (4–63 [0–9768])	0 (0–3 [0–596])	0 (0–4 [0–401])	3 (0–14 [0–1894])
Subgroup 1. Medical—Pharmaceutical intervention (*n* = 35)				
49 (8–123 [0–1173])	21 (4–86 [0–1145])	0 (0–3 [0–72])	0 (0–2 [0–26])	3 (1–13 [0–181])
Subgroup 2. Surgical interventions (*n* = 25)				
49 (17–76 [0–470])	33 (6–45 [0–184])	0 (0–2 [0–401])	0 (0–2 [0–47])	5 (0–10 [0–71])
Subgroup 3. Dental interventions (*n* = 6)				
79 (2–164 [0–1072])	36 (2–122 [0–800])	0 (0–18 [0–23])	0 (0–2 [0–3])	12 (0–60 [0–270])
Subgroup 4. Multidisciplinary health—Nutritional interventions (*n* = 16)				
13 (6–65 [0–1772])	5 (2–20 [0–392])	0 (0–0 [0–596])	0 (0–12 [0–307])	3 (0–11 [0–1464])
Subgroup 5. Multidisciplinary health—Physiotherapy interventions (*n* = 10)				
104 (21–1734 [0–2393])	21 (7–1291 [0–2126])	0 (0–11 [0–277])	17 (3–45 [0–87])	10 (2–120 [0–237])
Subgroup 6. Multidisciplinary health—Psychology interventions (*n* = 15)				
104 (61–569 [8–12,063])	61 (40–326 [5–9768])	1 (0–28 [0–401])	3 (0–11 [0–401])	12 (3–128 [0–1894])
Subgroup 7. Traditional Chinese medicine—Herbal and non‐herbal interventions (*n* = 9)				
0 (0–2 [0–39])	0 (0–0 [0–15])	0 (0–0 [0–0])	0 (0–0 [0–2])	0 (0–1 [0–24]

After removing 11 reviews that exceeded the 500‐records screening threshold, we screened a total of 6396 records (3871 from ClinicalTrials.gov, 924 from EudraCT, 658 from ANZCTR, and 943 from the WHO ICTRP search portal) across the 101 included reviews. The median (IQR) number of records screened per review was 30 (7–87) records from all registers; 13 (3–47) from ClinicalTrials.gov, 0 (0–5) from EudraCT, 0 (0–1) from ANZCTR, and 3 (0–10) from the WHO ICTRP search portal.

We identified a total of 282 trials (either with or without results available) meeting the eligibility criteria of the reviews and measuring any of its outcomes of interest for 63 out of 101 (63%) reviews. Fifty‐four reviews had at least one trial meeting the eligibility criteria without any trial results available, 34 reviews had at least one new eligible trial with trial results available, but not necessarily for inclusion in any of the meta‐analyses, and only 20 reviews had at least one new trial meeting the review eligibility criteria, with trial results able to be included in one of the original meta‐analyses (Table 3). The median (IQR [range]) number of eligible trials that measured any of the outcomes of interest to the review (regardless of whether results were available or not) identified per systematic review was 1 (0–4 [0–23]). Furthermore, this median varied from a register to another; 1 (0–2 [0–17]) for ClinicalTrials.gov, 0 (0–0 [0–3]) for EudraCT, 0 (0–0 [0–6]) for ANZCTR, and 0 (0–1 [0–13]) for the WHO ICTRP search portal. Moreover, there was a difference in these frequencies across different intervention subgroups (Table 3).

**TABLE 3:** Frequency (%) of systematic reviews with new trials identified, sub‐grouped according to intervention type (*n* = 101)

Type of systematic review	Had at least 1 new trial meeting the review eligibility criteria	Had at least 1 new trial meeting the review eligibility criteria, without any trial results available	Had at least 1 new trial meeting the review eligibility criteria, with trial results available, but not necessarily for inclusion in any of the meta‐analyses	Had at least 1 new trial meeting the review eligibility criteria, with trial results available for inclusion in one of the meta‐analyses
Overall results				
Frequency (%) of systematic reviews	63/101 (63%)	54/101 (53%)	34/101 (34%)	20/101 (20%)
Median (IQR [range]) of trials identified per systematic review)	1 (0–4 [0–23])	1 (0–3 [0–19])	0 (0–1 [0–17])	0 (0–0 [0–4])
Medical reviews—Pharmaceutical interventions				
Frequency (%) of systematic reviews	21/33 (64%)	17/33 (52%)	12/33 (36%)	8/33 (24%)
Median (IQR [range]) of trials identified per systematic review)	1 (0–4 [0–11])	1 (0–3 [0–6])	0 (0–1 [0–17])	0 (0–0 [0–3])
Medical reviews—Surgical interventions				
Frequency (%) of systematic reviews	15/25 (60%)	13/25 (52%)	5/25 (20%)	1/25 (4%)
Median (IQR [range]) of trials identified per systematic review)	1 (0–2 [0–13])	1 (0–3 [0–9])	0 (0–0 [0–4])	0 (0–0 [0–2])
Dental reviews—Dental interventions				
Frequency (%) of systematic reviews	4/5 (80%)	4/5 (80%)	3/5 (60%)	2/5 (40%)
Median (IQR [range]) of trials identified per systematic review)	4 (1–4 [0–6])	1 (0–3 [0–6])	2 (0–3 [0–4])	0 (0–3 [0–4])
Multidisciplinary health reviews—Nutritional interventions				
Frequency (%) of systematic reviews	11/14 (79%)	9/14 (64%)	4/14 (29%)	3/14 (21%)
Median (IQR [range]) of trials identified per systematic review)	2 (1–4 [0–7])	2 (0–4 [0–6])	0 (0–1 [0–5])	0 (0–0 [0–1])
Multidisciplinary health—Physiotherapy interventions				
Frequency (%) of systematic reviews	1/7 (86%)	6/7 (86%)	4/7 (57%)	3/7 (43%)
Median (IQR [range]) of trials identified per systematic review)	5 (2–10 [0–23)	2 (1–9 [0–19])	1 (0–2 [0–2])	1 (0–2 [0–2])
Multidisciplinary health—Psychology interventions				
Frequency (%) of systematic reviews	8/12 (67%)	7/12 (58%)	6/12 (50%)	3/12 (25%)
Median (IQR [range]) of trials identified per systematic review)	2 (0–9 [0–22])	0 (0–7 [0–19])	1 (0–3 [0–7])	0 (0–1 [0–2])
Traditional Chinese medicine—Herbal and non‐herbal interventions				
Frequency (%) of systematic reviews	1/9 (11%)	1/9 (11%)	1/9 (11%)	1/9 (11%)
Median (IQR [range]) of trials identified per systematic review)	0 (0–0 [0–8])	0 (0–0 [0–4])	0 (0–0 [0–4])	0 (0–0 [0–1])

Of the 20 reviews for which two authors independently screened trial registration records, there was at least one discrepancy in the number of trials deemed to meet the eligibility criteria for the review for 25% of reviews. However, after discussing the discrepancies, we determined that the first screener (who screened records for the remaining 80% of reviews) needed to correct their screening judgements for only 5% of reviews. Therefore, we anticipate the error rate in screening decisions for the reviews which were screened by one investigator to be 5%.

### 3.3 Re‐analyses of meta‐analysis results

**FIGURE 2:**
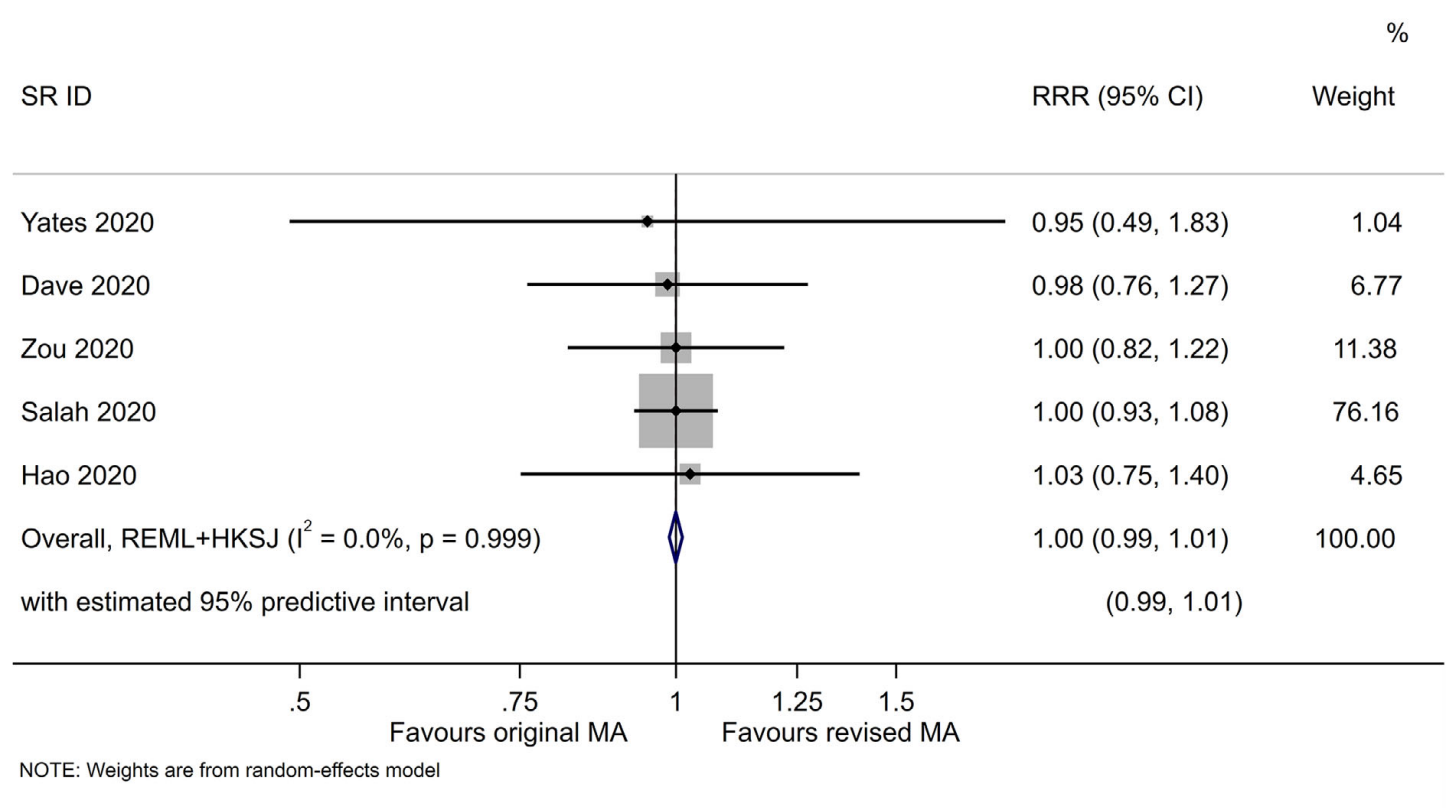
Random‐effects meta‐analysis (MA) of the difference in meta‐analytic risk ratios (ratio of risk ratios [RRR]) before versus after adding the new trials. RRR estimates below 1 indicate that the original meta‐analytic estimate was more favourable to the experimental intervention than the revised meta‐analytic estimate [Colour figure can be viewed at wileyonlinelibrary.com]

**FIGURE 3:**
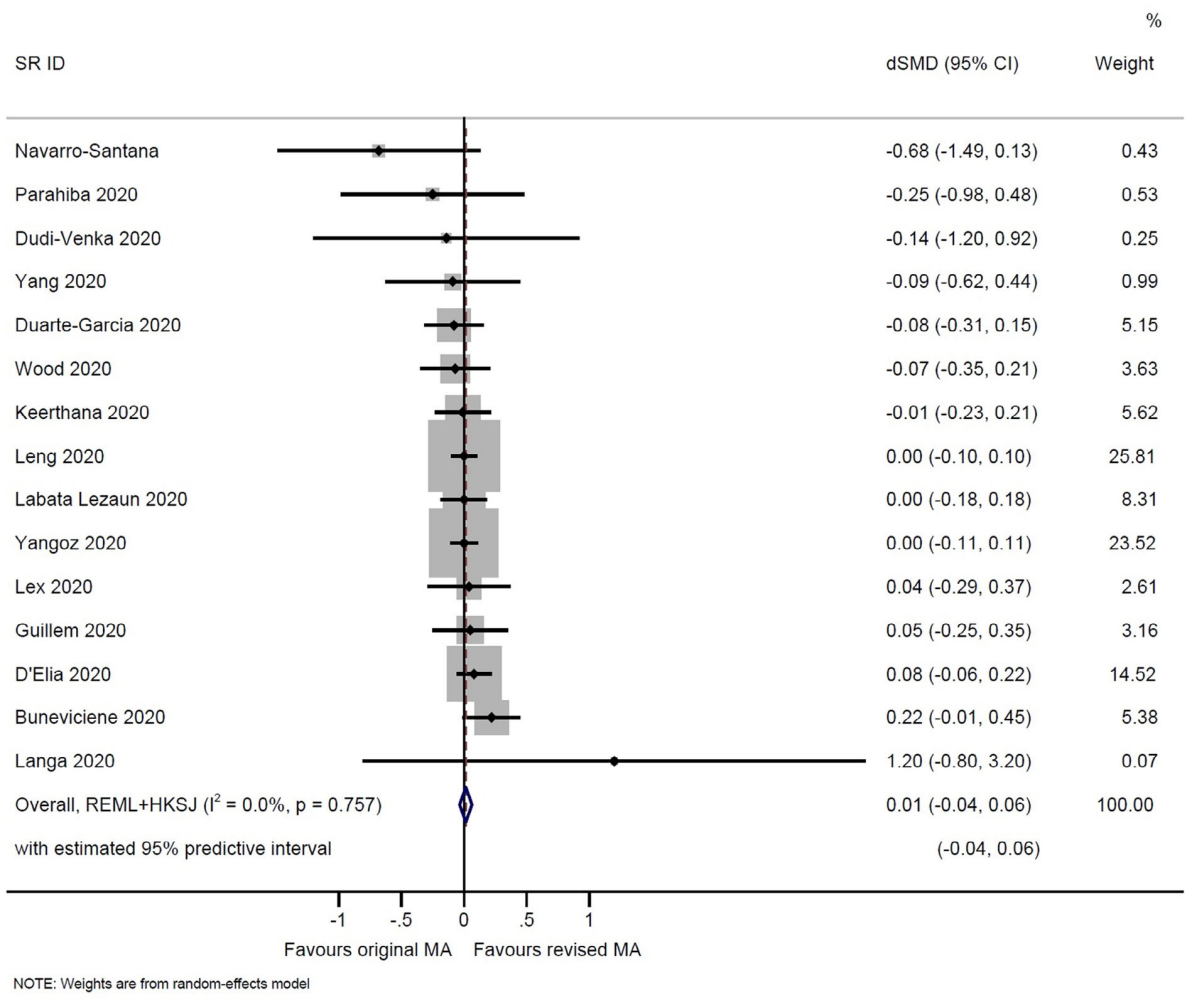
Random‐effects meta‐analysis (MA) of the difference in meta‐analytic standardised mean differences (dSMD) before versus after adding the new trials. dSMD estimates below 0 indicate that the original meta‐analytic estimate was more favourable to the experimental intervention than the revised meta‐analytic estimate [Colour figure can be viewed at wileyonlinelibrary.com]

A total of 29 new trials were included in the re‐analysis of a meta‐analysis in 20 systematic reviews, with a median of 2 trials (IQR 1–2 [range 1–4]) added per review. Outcome results for 18 trials were extracted from trials register records. Outcome results for the remaining 11 trials were extracted from trial publications which were published before the reviews' search dates. The weight of the additional trials included in the re‐analysis ranged from 0% to 36% (median 10%) (Table 4; Data S4 presents forest plots for the 20 meta‐analyses that we reanalysed). On average, there was no difference in the meta‐analytic risk ratios before versus after adding the new trials (combined RRR 1.00, 95% confidence interval 0.99–1.01; *I* ^2^ 0%; 95% predictive interval 0.99–1.01; based on 5 meta‐analyses of binary outcomes) (Figure 2). Similarly, there was no difference in the meta‐analytic standardised mean differences before versus after adding the new trials, on average (combined dSMD 0.01, 95% confidence interval −0.04 to 0.06; *I* ^2^ 0%; 95% predictive interval −0.04 to 0.06; based on 15 meta‐analyses of continuous outcomes) (Figure 3).

**TABLE 4:** Effects of adding new trials to the meta‐analyses (*n* = 20)

SR	Number of trials in original SR	Number of new trials meeting review eligibility criteria	Number of new trials with results that could contribute to a meta‐analysis	Outcome	Summary effect estimate in original SR	Summary effect estimate with new trials included	Weight of new trials included in selected meta‐analysis (%)
Yangoz 2020	14	49	1	Adherence to fluid intake	SMD −0.22 (−0.33, −0.11)	SMD −0.22 (−0.33, −0.11)	0
Wood 2020	4	5	1	Pressure pain sensitivity for non‐specific neck pain	SMD 0.40 (0.12, 0.68)	SMD 0.33 (0.06, 0.61)	0
Duarte‐Garcia 2020	5	1	1	Systemic lupus erythematosus (SLE) disease activity	SMD −0.33 (−0.56, −0.09)	SMD −0.25 (−0.45, −0.04)	30
Parahiba 2020	8	9	2	Lean body mass (kg)	MD 2.56 (1.26, 3.85)	MD 2.48 (1.39, 3.58)	13
Navarro‐Santana 2020	6	23	3	Short term pain	MD −2.31 (−3.63, −0.99)	MD −1.24 (−2.34, −0.15)	36
Labata Lezaun 2020	14	8	1	Lower limb strength	SMD 0.01 (−0.18, 0.19)	SMD 0.01 (−0.16, 0.17)	11
Leng 2020	11	12	1	Depressive symptoms	SMD −0.21 (−0.31, −0.10)	SMD −0.21 (−0.31, −0.11)	1
Lex 2020	3	12	1	Pain at rest on postop day 0	MD −1.37 (−2.28, −0.46)	MD −1.47 (−2.27, −0.67)	12
Dudi‐Venka 2020	5	6	1	Time to passage of stool	MD −0.83 (−1.31, −0.35)	MD −0.78 (−1.21, −0.36)	16
Langa 2020	9	4	3	The interproximal plaque index	MD 0.70 (0.57, 0.83)	MD 0.33 (0.10, 0.55)	24
Yang 2020	13	8	1	Six‐minute walk distance	MD 41.54 (28.53, 54.55)	MD 38.77 (26.25, 51.29)	8
Guillem 2020	7	3	1	Systolic blood pressure (mmHg)	MD −1.07 (−3.18, 1.04)	MD −1.48 (−3.61, 0.65)	8
D'Elia 2020	26	5	1	Systolic blood pressure (mmHg)	MD −3.14 (−4.43, −1.85)	MD −3.65 (−5.19, −2.11)	12
Buneviciene 2020	6	35	2	Health‐related quality of life (HRQoL)	SMD 0.27 (0.04, 0.50)	SMD 0.49 (0.04, 0.94)	25
Keerthana 2020	8	7	1	Rate of orthodontic movement of a maxillary canine tooth being distalised to close an extraction space	MD 0.34 (0.25, 0.42)	MD 0.33 (0.25, 0.41)	2
Hao 2020	8	4	1	Complete response rate	OR 35.82 (14.98, 85.64)	OR 37.56 (16.28, 86.70)	6
Salah 2020	8	12	2	All‐cause mortality	RR 0.84 (0.78, 0.91)	RR 0.84 (0.78, 0.91)	0
Zou 2020	10	12	3	Urinary tract infections (UTI)	RR 1.22 (1.00, 1.49)	RR 1.22 (1.00, 1.49)	1
Yates 2020	18	29	3	Overall venous thromboembolism (VTE) events	RR 0.74 (0.38, 1.42)	RR 0.78 (0.42, 1.45)	9
Dave 2020	14	5	1	Incidence of hepatocellular carcinoma (HCC)	RR 1.28 (0.99, 1.66)	RR 1.30 (1.01, 1.68)	1

Abbreviations: MD, mean difference; OR, odds ratio; RR, risk ratio; SMD, standardised mean difference; SR, systematic review.

## 4 DISCUSSION

### 4.1 Summary of the results

We searched and screened records from four clinical trial registers (ClinicalTrials.gov, EudraCT, ANZCTR, and WHO ICTRP search portal) to look for additional trials for 101 reviews that had not performed any clinical trials register search. We found additional trials that met the review eligibility criteria for 63% (63/101) of reviews; however, only 20% (20/101) of reviews had additional trials that could contribute to at least one meta‐analysis. When we re‐analysed a meta‐analysis in each of these 20 reviews, including the new trial results led to no change in the meta‐analytic estimates, on average.

### 4.2 Comparing to other studies

Our results are consistent with previous studies. Several studies from different medical specialties investigated the prevalence of clinical trials register searches in published systematic reviews, and results indicated that clinical trials register searches are underutilised by systematic reviewers.^7^, ^8^, ^9^, ^10^, ^11^, ^12^, ^13^, ^14^, ^15^, ^16^ In our study, 23% of reviews considering randomised (or randomised and non‐randomised studies) as eligible for inclusion conducted a search of a trials register. The equivalent percentage observed in other studies ranges from 6% to 35%.^7^, ^8^, ^9^, ^10^, ^11^, ^13^, ^14^, ^15^, ^16^, ^24^

Our findings also agree with two studies which examined the number of trials identified via ClinialTrials.gov for systematic reviews of surgical interventions.^12^, ^14^ Fuller et al. found additional eligible trials for 64% (16/25) of the reviews,^12^ while Reddy et al. found additional eligible studies for 59% (59/100) of reviews.^14^ Similarly, we identified additional trials for 60% (15/25) of the reviews of surgical interventions included in our study from different surgical subspecialities; mostly from orthopaedics surgery, followed by general surgery, neurosurgery, and bariatric surgery.

Apart from the case study by Wilson et al.,^19^ Baudard et al. was the only other study we are aware of to quantify the impact of searching trials registers on meta‐analyses. They examined only systematic reviews of pharmaceutical interventions (regardless of the health field), and they included data from additional trials for 14/95 reviews and found it did not change the statistical significance of meta‐analyses in any review.^18^ Similarly, we included data from additional trials for 20/101 reviews, and we observed no change in the meta‐analytic estimates, on average.

### 4.3 Potential explanations for the findings

There are several possible reasons why we identified additional trials with results for inclusion in only a minority of the 101 systematic reviews of pharmaceutical and non‐pharmaceutical interventions included in our evaluation. Firstly, despite the ICMJE mandating registration of all clinical trials, registration rates of clinical trials are still low for non‐pharmaceutical interventions, which are not subjected to the FDAAA regulatory rules that are limited to drug, biologic, or device interventions.^25^, ^26^, ^27^ Azar et al. conducted a cross‐sectional study of 953 trials for non‐regulated health care interventions published in 2016 and 2017 in 254 journals, and found that 55% of rehabilitation intervention trials, 52% of surgery trials, 48% of psychology or behavioural sciences trials, and 43% of nutrition or dietetics trials, had not been registered.^26^ Fifty‐eight percent of our included reviews addressed these non‐pharmaceutical interventions. Furthermore, despite the United States Food and Drug Administration Amendments Act (FDAAA) 2007 requiring sponsors of drug and device trials to report their results directly onto ClinicalTrials.gov within 1 year of completion, compliance is poor, and there is a lack of enforcement by regulators.^28^ This suggests more needs to be done to enforce registration of clinical trials and posting of trial results for trials of pharmaceutical and non‐pharmaceutical interventions.

The lack of change, on average, in the meta‐analytic estimates after including the additional trial results can be attributed to various factors. The magnitude of effect of the additional trials may not have differed considerably from the magnitude of effect of trials already included, leaving little opportunity for a deviation in the meta‐analytic effect. Furthermore, the limited number of additional trials identified, despite our comprehensive effort to search for them, means the ability to shift the meta‐analytic effects was itself limited. Our results should therefore be considered reflective of how clinical trials registers are currently used by trialists. We cannot rule out the possibility of greater value from searching trials registers under ideal conditions (i.e., when all trials are registered, and results of all trials are posted in the register in a timely manner).

### 4.4 Strengths and limitations

Our study has several strengths. It is the first study that evaluated the impact of searching clinical trial registers on meta‐analyses of pharmaceutical *or* non‐pharmaceutical health interventions, and we further classified non‐pharmaceutical interventions into sub‐categories to help clarify the picture. Moreover, our sample of included systematic reviews was taken from the REPRISE project, which was underpinned by a comprehensive search strategy and included a broad spectrum of reviews of pharmaceutical and non‐pharmaceutical health interventions. We searched four clinical trial registers; previous studies only searched ClinicalTrials.gov or the WHO ICTRP search portal. Furthermore, the search strategies we ran were adjusted individually for each of the trials registers to try to capture as many trials as possible; in some cases, we had to run the search up to four times per register to include every possible term, then we combined the records and removed duplicates. Also, we looked for conference abstracts to try to identify any useful data that could contribute to the meta‐analyses if a link or note to that abstract was provided in the register and no publications were found. All data extraction and reanalyses of meta‐analyses performed by the first author was checked for accuracy by a second author. Finally, we are the first to adopt the meta‐meta‐analytic approach to assess the difference in the effect estimates before and after adding the new trials, in this context.

Our study also has some limitations. We cannot rule out the possibility that some of the systematic reviewers might have conducted a search of a clinical trials register, yet did not report the search in the paper, because they perceived it was not helpful to do so. The search strategies we used in the trials registers were informed by the search strategies for bibliographic databases which were reported in the original reviews. It is possible that some of these original search strategies were not sufficiently comprehensive, and therefore additional eligible trials might have been identified had alternative search strategies been used in the trials registers. Also, some searches were not sufficiently specific, in that they yielded an infeasible number of records to go through. We only screened trials registration records if there were no more than 500 records identified per register per review, due to time and resources limitations; therefore, it is possible that additional eligible trials could be retrieved for those 11 reviews excluded which exceeded this threshold (most were psychology and physiotherapy reviews with a broad review question, and so our findings may have less applicability to such reviews). Furthermore, we searched Google for eligible trials without results posted in the register; however, we are unaware if searching PubMed or other databases instead could have retrieved publications for these trials.

Other limitations are that we are not experts in all the topics covered by the systematic reviews included, and thus it is possible that we might have misinterpreted the review eligibility criteria for some reviews, and hence misclassified some of the additional trials identified. Due to our limited resources, we did not contact the authors of the original reviews to confirm whether they would have included in their meta‐analyses the additional trials we identified (such trials might not have met some unreported inclusion criteria set by the review authors). In the interests of time, we chose not to screen the ClinicalTrials.gov, EudraCT and ANZCTR records identified in the WHO ICTRP searches, as we assumed this would be duplicative of our searches of these three registers. However, it is possible that we missed some eligible trials by following this approach. Furthermore, screening of trials registration records was largely done by one author; however, the error rate identified from the random sample of reviews for which double screening of trials registration records was done was sufficiently low (5%) that we are confident in our findings. For reasons of feasibility, we evaluated the impact of including data from registered trials on only one meta‐analysis per systematic review; it is possible that the trials we identified in the trials registers could have contributed to other meta‐analyses or syntheses in the reviews. We excluded systematic reviews that did not report a search strategy or those with no search dates, and so our sample might better reflect high‐quality systematic reviews only, hence limiting our findings' generalizability. Finally, we consider our sample size of 20 reviews as small to reflect the true impact of searching trials registers on meta‐analyses.

### 4.5 Implications for practice

Searching clinical trials registers could help identify additional trials to be included in a systematic review and is the currently the best way of estimating the true number of trials that have been conducted on a topic. For those registered trials with results available, systematic reviewers should consider including such results in their meta‐analyses, as there is no guarantee those results will be made publicly available in other formats. For registered trials without results posted, review authors should contact the trial team and invite them to supply any relevant results so that they can be included in the review. Furthermore, review authors can factor into consideration any registered trials with no available results in their assessments of the risk of reporting bias.^2^ In addition, sometimes trials register records provide information about methods trialists used, which may not appear in any other source, and so searching trials registers can provide a more complete picture about a trial.^29^ However, reviewers from different specialties should consider which registry to use, what search term adjustments might be required, and the time and resources necessary to run searches and screen records.

Cochrane recommends review authors search ClinicalTrials.gov and the WHO ICTRP at a minimum, an approach we agree with. The decision to search other registers should be influenced by the scope of the review. For example, in a surgical intervention review, searching ANZCTR could result in fewer records than searching ClinicalTrials.gov (median 0 (IQR 0–1 [range 0–47]) vs. 33 (6–45 [0–184])), whilst EudraCT is a register for pharmaceutical, not surgical interventions, and so all records retrieved from EudraCT are unlikely to be relevant to a review of a surgical intervention.

Moreover, searching clinical trial registers for some interventions, such as behavioural interventions and physiotherapy interventions, might require more time, as we observed that both review types yielded a larger number of records, when compared with our searches for reviews of medical pharmaceutical or surgical interventions (medians 104 (61–569 [8–12,063]) and 104 (21–1734 [0–2393]), vs. 49 (8–123 [0–1173]) and 49 (17–76 [0–470]), respectively, across the four registers). Furthermore, authors need to adjust the search strategy individually for each trial register, given the word limits, especially for ClinicalTrials.gov and ANZCTR; however, using a very long search strategy could lead to defaults or zero results in EudraCT and the WHO ICTRP search portal. Nevertheless, some registers' search options should be modified to facilitate searches conducted by systematic reviewers. For example, we faced some difficulties using the advanced search functions in the ANZCTR register and WHO ICTRP search portal, were unable to filter records in WHO ICTRP according to trial completion status (and so had to screen each record at the register to determine status), and we had to manually screen each ANZCTR and WHO ICTRP record to determine whether it had results available or not (given there is no option to filter the search according to availability of results), which required extra time and effort.

## 5 CONCLUSION

Searching clinical trials registers can help identify new eligible trials that match the eligibility criteria of the systematic review; however, currently many of the trials identified in trials registers are unlikely to have results available for inclusion in meta‐analyses. Furthermore, even if results are available for these registered trials, little or no difference might be found in the meta‐analytic effect estimates after including them in meta‐analyses. Trials registers would be even more valuable to systematic reviewers if more trialists made use of them (i.e., registered their trials and posted their results in a timely manner).

## AUTHOR CONTRIBUTIONS

All authors declare to meet the ICMJE conditions for authorship. Matthew J. Page designed the study. Zainab Alqaidoom developed the search strategies for trials registers. Zainab Alqaidoom, Maryam Awadh, Phi‐Yen Nguyen and Matthew J. Page screened trials register records. Zainab Alqaidoom, Phi‐Yen Nguyen and Matthew J. Page collected data from systematic reviews. Zainab Alqaidoom and Matthew J. Page collected data from trials register records. Zainab Alqaidoom and Matthew J. Page ran all analyses. Zainab Alqaidoom wrote the first draft of the article. All authors contributed to revisions of the article. All authors approved the final version of the submitted article.

## CONFLICT OF INTEREST

The authors declare no competing interests in relation to this study.

## FUNDING INFORMATION

Matthew J. Page is supported by an Australian Research Council Discovery Early Career Researcher Award (DE200101618). The funders had no role in the study design, decision to publish, or preparation of the manuscript.

## Supporting information


**APPENDIX S1** Supporting Information

## Data Availability

Data and analytic code are available on the Open Science Framework (https://osf.io/y3gzv/).
