# Supplementary material for: Impact of searching clinical trials registers in systematic reviews of pharmaceutical and non‐pharmaceutical interventions: Reanalysis of meta‐analyses
Source: Res Synth Methods. 2022 Jul 28;14(1):52–67. doi: 10.1002/jrsm.1583 (PMC10087877; doi:10.1002/jrsm.1583)
Supplement: Supplementary file 1 — APPENDIX S1 Supporting Information [file JRSM-14-52-s001.docx]

**Supplementary Material**

**Impact of searching clinical trials registers in systematic reviews of pharmaceutical and non-pharmaceutical interventions: reanalysis of meta-analyses**

Zainab Alqaidoom^1^, Phi-Yen Nguyen^1^, Maryam Awadh^2^, Matthew J. Page^1^

1. School of Public Health and Preventive Medicine, Monash University, Melbourne, Australia
2. School of Medicine, Southeast University, Nanjing, China

**Correspondence to:** Dr. Matthew Page, School of Public Health and Preventive Medicine,

Monash University, 553 St Kilda Road, Melbourne, Victoria, 3004, Australia. Telephone: +61 9903 0248. Email address: [matthew.page@monash.edu](mailto:matthew.page@monash.edu)

**Contents**

[Supplementary file 1. Search strategies to identify systematic reviews 3](#_Toc89784631)

[Supplementary file 2. Steps we followed to search each register 4](#_Toc89784632)

[Supplementary file 3. Trials register search strategies we used for each systematic review 6](#_Toc89784633)

[Supplementary file 4. Impact of trial registers searches on summary estimates of meta-analyses 61](#_Toc89784634)

## **Supplementary file 1. Search strategies to identify systematic reviews**

Searches run Thursday 3^rd^ December 2020

**PubMed**

(meta-analysis[PT] OR meta-analysis[TI] OR systematic[sb]) AND 2020/11/02:2020/12/02[EDAT]

**Science Citation Index Expanded (SCI-EXPANDED) and Social Sciences Citation Index (SSCI) via Web of Science**

(TI=meta-analysis OR AB=meta-analysis OR TS=meta-analysis OR TI="systematic review" OR AB="systematic review" OR TS="systematic review") AND **LANGUAGE**: (English) AND **DOCUMENT** **TYPES**: (Article OR Review)

*Indexes=SCI-EXPANDED, SSCI; Timespan=Last 4 weeks*

**Scopus via Elsevier**

TITLE("meta-analysis" OR "systematic review") AND ORIG-LOAD-DATE > 1604275200 AND ORIG-LOAD-DATE < 1606867200 AND (LIMIT-TO(DOCTYPE, "ar") OR LIMIT-TO(DOCTYPE, "re"))

**Education Collection via ProQuest (added in 30 last days)**

MAINSUBJECT.EXACT.EXPLODE("Meta Analysis") OR ab(meta-analysis OR systematic review) OR ti(meta-analysis OR systematic review)

## **Supplementary file 2. Steps we followed to search each register**

**1. Clinicaltrials.gov:**

- The advanced search function was used.
- We specified population terms in the "condition/disease" and intervention terms in the "other terms" section.
- We applied the following filters: Study type: Interventional studies, and Status: "recruitment completed".
- The search was performed for trials with and without results separately.
- All records identified were exported by selecting "all available columns" into a "comma-separated values" format and then importing the data into our ClinicalTrials.gov Excel screening sheet.
- Once in Excel, records were sorted by "completion date" (or if not available, by "primary completion date") for initial screening; studies completed after the date in which systematic reviewers had conducted their search were excluded from our counts.
- Publication links attached to the record were also checked to identify any publications, especially for trials without results posted in the register.
- Screening outputs were recorded in the register screening sheet and the overall data extraction sheet.

**2. EudraCT:**

- In the basic search toolbar, the search strategy was entered using the Boolean operators: (Condition OR Condition) AND (intervention OR intervention).
- In the advanced search, trial status selected was: completed.
- All trials with and without results’ summary details were exported separately in a “plain text format” unless there were a total of fewer than 5 trials in one group, whereby we imported them together (records with and without results).
- A maximum of 20 records/page can be imported, and all records were imported into our 1 EudraCT screening sheet.
- Records were screened manually by the start date. All start dates should be before the date in which the systematic reviewers had conducted their search.
- For records with results, all/some centres should be marked as “completed”, and if some centres were marked “prematurely ended” or “terminated” or “ongoing” for the same record, the overall trial record and results were screened.
- For records without results, if any centre was marked “ongoing”, we considered the overall trial uncompleted, and the record was excluded from screening.
- Details for each record were accessed through the link provided for further screening (participants, interventions, and outcomes).
- For trials without results that are eligible and which reported outcomes of interest to the systematic reviewers, we searched Google to identify if any publications were available but details not updated in the EudraCT record.
- Eligibility and screening outputs were reported into our EudraCT screening sheet and the main data extraction sheet.
- For eligible trials that measured the review outcome of interests and no results were updated in the register, we performed a quick Google search by using the trial title to try to detect if any publications were available, and used the following variables to confirm similarity: authors names, affiliations, funding source, and abstract or methods.

**3. ANZCTR:**

- Similar to EudraCT, the search strategy was performed using the Boolean operators.
- The advanced search options were used for the following inputs only: registry: ANZCTR, study type: interventional, allocation to intervention: randomised, and recruitment status: completed.
- All records identified were exported via “Excel file” format, and we copied the following variables: “Trial id, ACTRN, submit date, and scientific title” into our ANZCTR screening sheet.
- Trials were sorted and screened according to the “registration date”. Any trial that was registered after the date in which the systematic reviewers conducted their search was excluded.
- Each trial was screened manually by copying its id in the ANZCTR basic search online, then assessing it against the review eligibility criteria.
- We looked for publication status on the record page and performed an additional Google search for trials that met the review eligibility criteria and measured outcomes of interest to the review, but which were marked as having no publications available in the register.
- We recorded eligibility and results-available outputs in our ANZCTR screening sheet and the main data extraction sheet.
- For each trial that matched the eligibility criteria and measured outcomes of interest of the reviews with no results links or comments available, we did a quick Google search to identify any for potential publications. The title of each trial was used for the search and we checked the authors names, affiliations, funding source, and abstract or methods to confirm similarity.

**4. WHO ICTRP search portal:**

- Similar to EudraCT and ANZCTR, the basic search function was used using the Boolean operators.
- Records were exported to CSV files and then imported into our 1 WHO ICTRP screening sheet.
- Records were filtered initially by “TrialID” to excluded trials from ANZCTR, ClinicalTrials.gov, and EudraCT. Then records were sorted by “date registration”, and trials with a registration date after the date in which the systematic reviewers had conducted their search were excluded.
- The screening was performed from original trial registers, and “status” was checked first in each register to check if the trial was “completed” or not. Ongoing trials or trials completed after the date of the last search for the systematic review were excluded. We also checked whether results were posted or publications attached in each register, and for trials meeting the review eligibility criteria and measuring outcomes of interest to the review, we searched Google to look for any potential publications using the title of each record, and checking the authors names, affiliations, funding source, and abstract or methods to confirm similarity.
- We recorded trial eligibility and results status in the WHO ICTRP screening sheet and our main data extraction sheet.

## **Supplementary file 3. Trials register search strategies we used for each systematic review**

**1.Shen_2020: 5-HT(3)receptor antagonists for the prevention of perioperative shivering undergoing spinal anaesthesia: a systematic review and meta-analysis of randomised controlled trials**

**CT.GOV**

(postoperative period) AND (5- HT3Ras OR serotonin OR 5-ht3 OR ondansetron OR ramosetron OR palonosetron OR granisetron OR tropisetron OR dolasetron OR alosetron)

**EDURACT**

(postoperative period) AND (5- HT3Ras OR serotonin OR 5-ht3 OR ondansetron OR ramosetron OR palonosetron OR granisetron OR tropisetron OR dolasetron OR alosetron)

**ANZCTR**

(postoperative period) AND (5- HT3Ras OR serotonin OR 5-ht3 OR ondansetron)

(postoperative period) AND (ramosetron OR palonosetron OR granisetron)

(postoperative period) AND ( tropisetron OR dolasetron OR alosetron)

**WHO ICTRP**

10 records for 10 trials found for: (postoperative period) AND (5- HT3Ras OR serotonin OR 5-ht3 OR ondansetron OR ramosetron OR palonosetron OR granisetron OR tropisetron OR dolasetron OR alosetron)

**2.Hao_2020: A meta-analysis of efficacy of topical steroids in eosinophilic esophagitis: From the perspective of histologic, clinical, and endoscopic outcome**

**CT.GOV**

(eosinophilic esophagitis) AND (topical steroid OR topical steroids OR budesonide OR fluticasone OR topical corticosteroid OR topical corticosteroids)

**EDURACT**

(eosinophilic esophagitis) AND (topical steroid OR topical steroids OR budesonide OR fluticasone OR topical corticosteroid OR topical corticosteroids)

**ANZCTR**

(eosinophilic esophagitis) AND (topical steroid OR budesonide OR fluticasone OR corticosteroid)

**WHO ICTRP**

(eosinophilic esophagitis) AND (topical steroid OR topical steroids OR budesonide OR fluticasone OR topical corticosteroid OR topical corticosteroids)

**3.Li_2020: An evaluation of the effects and safety of Zuogui pill for treating osteoporosis: Current evidence for an ancient Chinese herbal formula**

**CT.GOV**

(osteoporosis OR primary osteoporosis OR postmenopausal osteoporosis OR senile osteoporosis OR age related osteoporosis OR bone loss OR osteopenia) AND (Zuogui Pill OR Zuogui granules)

**EDURACT**

(osteoporosis OR primary osteoporosis OR postmenopausal osteoporosis OR senile osteoporosis OR age related osteoporosis OR bone loss OR osteopenia) AND (Zuogui Pill OR Zuogui granules)

**ANZCTR**

(osteoporosis OR bone loss OR osteopenia) AND (Zuogui Pill OR Zuogui granules)

**WHO ICTRP**

(osteoporosis OR primary osteoporosis OR postmenopausal osteoporosis OR senile osteoporosis OR age related osteoporosis OR bone loss OR osteopenia) AND (Zuogui Pill OR Zuogui granules)

**4.Jakubczyk_2020: Antioxidant Potential of Curcumin-A Meta-Analysis of Randomized Clinical Trials**

**CT.GOV**

(antioxidant OR antioxidant agent OR antioxidant nutrient OR antioxidants OR antioxidation agent OR antioxidation product OR antioxidative OR antioxidant OR phenolic antioxidant) AND (curcumin OR curcumine OR turmeric yellow)

**EDURACT**

(antioxidant OR antioxidant agent OR antioxidant nutrient OR antioxidants OR antioxidation agent OR antioxidation product OR antioxidative OR antioxidant OR phenolic antioxidant) AND (curcumin OR curcumine OR turmeric yellow)

**ANZCTR**

(Antioxidant OR antioxidative agent) AND (Curcumin OR Turmeric yellow)

**WHO ICTRP**

(antioxidant OR antioxidant agent OR antioxidant nutrient OR antioxidants OR antioxidation agent OR antioxidation product OR antioxidative OR antioxidant OR phenolic antioxidant) AND (curcumin OR curcumine OR turmeric yellow)

**6.Arnold_2020: Brief report on a systematic review and meta-analysis of early childhood educational programming and teenage pregnancy prevention**

**CT.GOV**

(teen pregnancy) AND (children OR early childhood intervention)

**EDURACT**

(teen pregnancy) AND (children OR early childhood intervention)

**ANZCTR**

(teen pregnancy) AND (children OR early childhood intervention)

**WHO ICTRP**

(teen pregnancy) AND (children OR early childhood intervention)

**7.Tirupathi_2020: Buffered versus unbuffered local anesthesia for inferior alveolar nerve block injections in children: a systematic review**

**CT.GOV**

(buffering) AND (local anesthesia) AND (dental)

**EDURACT**

(buffering) AND (local anesthesia) AND (dental)

**ANZCTR**

(buffering) AND (local anesthesia) AND (dental)

**WHO ICTRP**

(buffering) AND (local anesthesia) AND (dental)

**8.Silva_2020: Cervical dilatation and preparation prior to outpatient hysteroscopy: A systematic-review and meta-analysis**

**CT.GOV**

(hysteroscopy) AND (cervical preparation OR cervical ripening OR cervical dilatation OR outpatient OR office OR ambulatory)

**EDURACT**

(hysteroscopy) AND (cervical preparation OR cervical ripening OR cervical dilatation OR outpatient OR office OR ambulatory)

**ANZCTR**

(hysteroscopy) AND (cervical preparation OR cervical ripening OR cervical dilatation)

**WHO ICTRP**

(hysteroscopy) AND (cervical preparation OR cervical ripening OR cervical dilatation OR outpatient OR office OR ambulatory)

**9.Lai_2021: Chinese herbal medicine (Rupi Sanjie capsule) for the treatment of breast pain: A systematic review and meta-analysis of randomized clinical trials**

**CT.GOV**

(breast pain OR breast fibrocystic disease OR breast hyperplasia OR mastopathy OR fibroadenosis OR breast fibroadenosis ) AND (Rupi Sanjie OR Chinese patent medicine )

**EDURACT**

(breast pain OR breast fibrocystic disease OR breast hyperplasia OR mastopathy OR fibroadenosis OR breast fibroadenosis ) AND (Rupi Sanjie OR Chinese patent medicine )

**ANZCTR**

(breast OR mastopathy OR breast fibroadenosis) AND (Rupi Sanjie OR Chinese patent medicine)

**WHO ICTRP**

(breast pain OR breast fibrocystic disease OR breast hyperplasia OR mastopathy OR fibroadenosis OR breast fibroadenosis ) AND (Rupi Sanjie OR Chinese patent medicine )

**10.Zeng_2020: Chinese Herbal Medicine Buyang Huanwu Decoction combined with acupuncture to treat sequela of apoplexy: a meta-analysis of randomized controlled trials**

**CT.GOV**

(Sequelae of stroke OR Sequela of apoplexy) AND (Buyang Huanwu Decoction OR Buyang Huanwu Decoction combined with acupuncture treatment OR Acupuncture OR Acupuncture and Moxibustion Treatment of Buyang Huanwu Decoction)

**EDURACT**

(Sequelae of stroke OR Sequela of apoplexy) AND (Buyang Huanwu Decoction OR Buyang Huanwu Decoction combined with acupuncture treatment OR Acupuncture OR Acupuncture and Moxibustion Treatment of Buyang Huanwu Decoction)

**ANZCTR**

(stroke OR Sequela of apoplexy) AND (Buyang Huanwu Decoction OR acupuncture OR Moxibustion)

**WHO ICTRP**

(Sequelae of stroke OR Sequela of apoplexy) AND (Buyang Huanwu Decoction OR Buyang Huanwu Decoction combined with acupuncture treatment OR Acupuncture OR Acupuncture and Moxibustion Treatment of Buyang Huanwu Decoction)

**11.Assis_2020: Clinical Efficiency of Self-etching One-Step and Two-Step Adhesives in NCCL: A Systematic Review and Meta-analysis**

**CT.GOV**

(Adhesive dental) AND (one-step AND two-step)

**EDURACT**

(Adhesive dental) AND (one-step AND two-step)

**ANZCTR**

(Adhesive dental) AND (one-step AND two-step)

**WHO ICTRP**

(Adhesive dental) AND (one-step AND two-step)

**12.Xu_2020: Combined Treatment of Non-Small-Cell Lung Cancer Using Shenyi Capsule and Platinum-Based Chemotherapy: A Meta-Analysis and Systematic Review**

**CT.GOV**

(non-small cell lung cancer OR Lung Carcinoma OR Non-Small-Cell OR Non-Small-Cell Lung Carcinoma OR NSCLC) AND ( Shenyi capsule AND Chemotherapy)

**EUDRACT**

(non-small cell lung cancer OR Lung Carcinoma OR Non-Small-Cell OR Non-Small-Cell Lung Carcinoma OR NSCLC) AND ( Shenyi capsule AND Chemotherapy)

**ANZCTR**

(non-small cell lung cancer OR NSCLC) AND ( Shenyi capsule AND Chemotherapy)

**WHO ICTRP**

(non-small cell lung cancer OR Lung Carcinoma OR Non-Small-Cell OR Non-Small-Cell Lung Carcinoma OR NSCLC) AND ( Shenyi capsule AND Chemotherapy)

**13.Yangoz_2020: Comparison of the Effect of Educational and Self-Management Interventions on Adherence to Treatment in Hemodialysis Patients: A Systematic Review and Meta-Analysis of Randomized Controlled Trials**

**CT.GOV**

(non-small cell lung cancer OR Lung Carcinoma OR Non-Small-Cell OR Non-Small-Cell Lung Carcinoma OR NSCLC) AND ( Shenyi capsule AND Chemotherapy)

**EUDRACT**

(non-small cell lung cancer OR Lung Carcinoma OR Non-Small-Cell OR Non-Small-Cell Lung Carcinoma OR NSCLC) AND ( Shenyi capsule AND Chemotherapy)

**ANZCTR**

(non-small cell lung cancer OR NSCLC) AND ( Shenyi capsule AND Chemotherapy)

**WHO ICTRP**

(non-small cell lung cancer OR Lung Carcinoma OR Non-Small-Cell OR Non-Small-Cell Lung Carcinoma OR NSCLC) AND ( Shenyi capsule AND Chemotherapy)

**14.Li_2020: Diabetic Kidney Disease Benefits from Intensive Low-Protein Diet: Updated Systematic Review and Meta-analysis**

**CT.GOV**

(Diabetic nephropathies OR diabetic nephropathy OR diabetic glomerulo OR diabetic kidney disease OR diabetic renal disease) AND (protein restricted diet OR protein restrict OR low protein)

**EUDRACT**

(Diabetic nephropathies OR diabetic nephropathy OR diabetic glomerulo OR diabetic kidney disease OR diabetic renal disease) AND (protein restricted diet OR protein restrict OR low protein)

**ANZCTR**

(diabetic nephropathy OR diabetic kidney OR diabetic renal) AND (protein restrict OR low protein)

**WHO ICTRP**

(Diabetic nephropathies OR diabetic nephropathy OR diabetic glomerulo OR diabetic kidney disease OR diabetic renal disease) AND (protein restricted diet OR protein restrict OR low protein)

**15.Wood_2020: Dry cupping for musculoskeletal pain and range of motion: A systematic review and meta-analysis**

**CT.GOV**

(musculoskeletal pain OR musculoskeletal diseases OR muscular disease OR myopathy OR myopathies OR myopathic condition OR muscle disorder OR myalgia OR myofascial pain OR myofascial pain syndrome OR acute pain OR chronic pain OR pain management) AND (dry cupping OR myofascial cupping OR myofascial decompression OR negative pressure cupping OR cupping therapy)

**EUDRACT**

(musculoskeletal pain OR musculoskeletal diseases OR muscular disease OR myopathy OR myopathies OR myopathic condition OR muscle disorder OR myalgia OR myofascial pain OR myofascial pain syndrome OR acute pain OR chronic pain OR pain management) AND (dry cupping OR myofascial cupping OR myofascial decompression OR negative pressure cupping OR cupping therapy)

**ANZCTR**

(musculoskeletal pain OR myopathy OR myofascial) AND (dry cupping OR cupping therapy)

**WHO ICTRP**

(Diabetic nephropathies OR diabetic nephropathy OR diabetic glomerulo OR diabetic kidney disease OR diabetic renal disease) AND (protein restricted diet OR protein restrict OR low protein)

**16.Griesinger_2020: Dydrogesterone as an oral alternative to vaginal progesterone for IVF luteal phase support: A systematic review and individual participant data meta-analysis**

**CT.GOV**

(luteal AND in vitro fertilization) OR (in vitro fertilization OR test tube fertilization OR IVF OR ICSI OR embryo OR blastocyst OR oocyte OR egg) AND

(dydrogesterone OR duphaston OR dabroston OR dufaston OR terolut OR isopregnenone OR dehydrogesterone) AND (progesterone OR progesterone OR progesterone)

**EUDRACT**

(luteal AND in vitro fertilization) OR (in vitro fertilization OR test tube fertilization OR IVF OR ICSI OR embryo OR blastocyst OR oocyte OR egg) AND (dydrogesterone OR duphaston OR dabroston OR dufaston OR terolut OR isopregnenone OR dehydrogesterone) AND (progesterone OR progesterone OR progesterone)

**ANZCTR**

(luteal AND in vitro fertilization) OR (IVF) AND (dydrogesterone) AND (progesterone)

**WHO ICTRP**

(luteal AND in vitro fertilization) OR (in vitro fertilization OR test tube fertilization OR IVF OR ICSI OR embryo OR blastocyst OR oocyte OR egg) AND (dydrogesterone OR duphaston OR dabroston OR dufaston OR terolut OR isopregnenone OR dehydrogesterone) AND (progesterone OR progesterone OR progesterone)

**19.Duarte-Garcia_2020: Effect of omega-3 fatty acids on systemic lupus erythematosus disease activity: A systematic review and meta-analysis**

**CT.GOV**

(Lupus OR libman sacks disease OR malignant dermatovisceritism OR osler libman sacks disease) AND

(Omega-3 Fatty Acid OR alpha-linolenic acid OR docosahexaenoate OR docosahexaenoic OR eicosapentaenoic acid OR eicosapentanoic OR fish oil OR linolenate OR linolenic acid)

**EUDRACT**

(Lupus OR libman sacks disease OR malignant dermatovisceritism OR osler libman sacks disease) AND (Omega-3 Fatty Acid OR alpha-linolenic acid OR docosahexaenoate OR docosahexaenoic OR eicosapentaenoic acid OR eicosapentanoic OR fish oil OR linolenate OR linolenic acid)

**ANZCTR**

(Lupus OR libman sacks OR lupus nephritis) AND (Omega 3 OR linolenic acid)

**WHO ICTRP**

(Lupus OR libman sacks disease OR malignant dermatovisceritism OR osler libman sacks disease) AND ( Omega-3 Fatty Acid OR alpha-linolenic acid OR docosahexaenoate OR docosahexaenoic OR eicosapentaenoic acid OR eicosapentanoic OR fish oil OR linolenate OR linolenic acid)

**20.Jazayeri_2020: Effect of omega-3 fatty acids supplementation on anthropometric indices in children and adolescents: A systematic review and meta-analysis of randomized controlled trials**

**CT.GOV**

(Childhood Obesity OR Adolescent Overweight) AND (Fish Oil OR (Omega-3 Fatty acids) OR n-3 Fatty Acids)

**EUDRACT**

(Childhood Obesity OR Adolescent Overweight) AND (Fish Oil OR (Omega-3 Fatty acids) OR n-3 Fatty Acids)

**ANZCTR**

(Childhood Obesity OR Adolescent Overweight) AND (Fish Oil OR Omega-3 Fatty Acids)

**WHO ICTRP**

(Childhood Obesity OR Adolescent Overweight) AND (Fish Oil OR (Omega-3 Fatty acids) OR n-3 Fatty Acids)

**22.Salah_2020: Effect of sodium-glucose cotransporter 2 inhibitors on cardiovascular and kidney outcomes-Systematic review and meta-analysis of randomized placebo-controlled trials**

**CT.GOV**

(Myocardial Infarction OR stroke OR death OR MACE OR major adverse cardiovascular events OR major adverse cardiac events OR heart failure) AND (Empagliflozin OR Dapagliflozin OR Canagliflozin OR Ertugliflozin)

**EUDRACT**

(Myocardial Infarction OR stroke OR death OR MACE OR major adverse cardiovascular events OR major adverse cardiac events OR heart failure) AND (Empagliflozin OR Dapagliflozin OR Canagliflozin OR Ertugliflozin)

**ANZCTR**

(Myocardial OR stroke) AND (Empagliflozin OR Dapagliflozin OR Canagliflozin OR Ertugliflozin)

(Cardiovascular OR heart) AND (Empagliflozin OR Dapagliflozin OR Canagliflozin OR Ertugliflozin)

**WHO ICTRP**

( Myocardial Infarction OR stroke OR death OR MACE OR major adverse cardiovascular events OR major adverse cardiac events OR heart failure) AND (Empagliflozin OR Dapagliflozin OR Canagliflozin OR Ertugliflozin)

**23.Parahiba_2020: Effect of testosterone supplementation on sarcopenic components in middle-aged and elderly men: A systematic review and meta-analysis**

**CT.GOV**

(Sarcopenia OR muscular OR muscle OR dystrophies OR atrophy OR neurotrophic) AND (Testosterone OR 17 beta Hydroxy 4 Androsten 3 one OR Androtop OR Histerone OR Sterotate OR Sustanon OR Androderm OR Testoderm OR Testolin OR Testopel OR Testosterone Sulfate OR AndroGel OR 8 Isotestosterone )

(Sarcopenia OR muscular OR muscle OR dystrophies OR atrophy OR neurotrophic) AND (17 beta Hydroxy 8 alpha 4 Androsten 3 one OR Andropatch OR Testim)

**EUDRACT**

(Sarcopenia OR muscular OR muscle OR dystrophies OR atrophy OR neurotrophic) AND (Testosterone OR 17 beta Hydroxy 4 Androsten 3 one OR Androtop OR Histerone OR Sterotate OR Sustanon OR Androderm OR Testoderm OR Testolin OR Testopel OR Testosterone Sulfate OR AndroGel OR 8 Isotestosterone OR 17 beta Hydroxy 8 alpha 4 Androsten 3 one OR Andropatch OR Testim)

**ANZCTR**

(Sarcopenia OR muscular OR muscle OR dystrophies OR atrophy OR) AND (Testosterone OR AndroGel)

**WHO ICTRP**

(Sarcopenia OR muscular OR muscle OR dystrophies OR atrophy OR neurotrophic) AND (Testosterone OR 17 beta Hydroxy 4 Androsten 3 one OR Androtop OR Histerone OR Sterotate OR Sustanon OR Androderm OR Testoderm OR Testolin OR Testopel OR Testosterone Sulfate OR AndroGel OR 8 Isotestosterone OR 17 beta Hydroxy 8 alpha 4 Androsten 3 one OR Andropatch OR Testim

**24.Navarro-Santana_2020: Effectiveness of Dry Needling for Myofascial Trigger Points Associated with Neck Pain Symptoms: An Updated Systematic Review and Meta-Analysis**

**CT.GOV**

(Cervical Pain OR Mechanical Neck Pain OR Myofascial Neck Pain) AND (Dry Needling OR Trigger Point Acupuncture OR Needling Therapy OR Intramuscular Stimulation)

**EUDRACT**

(Cervical Pain OR Mechanical Neck Pain OR Myofascial Neck Pain) AND (Dry Needling OR Trigger Point Acupuncture OR Needling Therapy OR Intramuscular Stimulation)

**ANZCTR**

(Cervical Pain OR Mechanical Neck Pain OR Myofascial Neck Pain) AND (Dry Needling OR Trigger Point Acupuncture OR Needling Therapy OR Intramuscular Stimulation)

**WHO ICTRP**

(Cervical Pain OR Mechanical Neck Pain OR Myofascial Neck Pain) AND (Dry Needling OR Trigger Point Acupuncture OR Needling Therapy OR Intramuscular Stimulation)

**26.Gonzalez-Medina_2020: Effectiveness of Global Postural Reeducation in Ankylosing Spondylitis: A Systematic Review and Meta-Analysis**

**CT.GOV**

(Global Postural Reeducation) AND:

(Spondylitis OR Spondylitis, Ankylosing OR Spondylarthropathies OR spondyloarthritis ankylopoietica OR ankylosing spondylarthritis OR ankylosing spondylarthritides OR spondylarthritides, ankylosing OR spondylarthritis, ankylosing)

---

(ankylosing spondylitis OR spondylarthritis ankylopoietica OR spondylitis ankylopoietica OR ankylosing spondyloarthritis OR ankylosing spondylarthritides OR spondylarthritides, ankylosing OR spondyloarthritis, ankylosing)

---

(bechterew disease OR bechterew’s disease OR bechterew s disease OR marie-struempell disease OR marie struempell disease OR rheumatoid spondylitis )

**EUDRACT**

(Global Postural Reeducation) AND(Spondylitis OR Spondylitis OR Ankylosing OR Spondylarthropathies OR spondyloarthritis ankylopoietica OR ankylosing spondylarthritis OR ankylosing spondylarthritides OR spondylarthritides OR bechterew OR marie-struempell disease OR marie struempell disease OR rheumatoid spondylitis)

**ANZCTR**

(ankylosing spondylarthritis OR ankylosing Spondylitis) AND (Global Postural Reeducation)

**WHO ICTRP**

(Global Postural Reeducation) AND(Spondylitis OR Spondylitis OR Ankylosing OR Spondylarthropathies OR spondyloarthritis ankylopoietica OR ankylosing spondylarthritis OR ankylosing spondylarthritides OR spondylarthritides OR bechterew OR marie-struempell disease OR marie struempell disease OR rheumatoid spondylitis)

**27.Labata-Lezaun_2020: Effectiveness of Protein Supplementation Combined with Resistance Training on Muscle Strength and Physical Performance in Elderly: A Systematic Review and Meta-Analysis**

**CT.GOV**

(Aged OR old people OR elderly OR aging OR older people OR older adults OR old adults OR frail OR senior OR geriatric) AND (Resistance training OR strength training) AND (Protein supplementation)

**EUDRACT**

(Aged OR old people OR elderly OR aging OR older people OR older adults OR old adults OR frail OR senior OR geriatric) AND (Resistance training OR strength training) AND (Protein supplementation)

**ANZCTR**

(Elderly OR old OR aged) AND (Resistance OR training) AND (Protein supplementation)

**WHO ICTRP**

(Aged OR old people OR elderly OR aging OR older people OR older adults OR old adults OR frail OR senior OR geriatric) AND (Resistance training OR strength training) AND (Protein supplementation)

**28.Li_2020: Effects of anesthesia depth on postoperative cognitive function and inflammation: a systematic review and meta-analysis**

**CT.GOV**

(Delirium OR cognitive dysfunc­tion OR POCD OR cognitive function OR cognition) AND (bispectral index OR bis OR narcotrend OR depth of anesthe­sia)

**EUDRACT**

(Delirium OR cognitive dysfunc­tion OR POCD OR cognitive function OR cognition) AND (bispectral index OR bis OR narcotrend OR depth of anesthe­sia)

**ANZCTR**

(delirium OR cognitive dysfunction) AND (bispectral index OR bis OR depth of anesthesia)

**WHO ICTRP**

(Delirium OR cognitive dysfunction OR POCD OR cognitive function OR cognition) AND (bispectral index OR bis OR narcotrend OR depth of anesthesia)

**30.Sarabon_2020 Effects of Resistance Exercise on Balance Ability: Systematic Review and Meta-Analysis of Randomized Controlled Trials**

**CT.GOV**

(star excursion OR Y-test OR Y test OR Y-balance test OR Romberg test OR single-leg stance OR functional reach test OR limits of stability OR limits-of-stability OR timed up and go) AND (resistance exercise OR strength training OR power training OR weight lifting OR muscle strengthening)

**EUDRACT**

(star excursion OR Y-test OR Y test OR Y-balance test OR Romberg test OR single-leg stance OR functional reach test OR limits of stability OR limits-of-stability OR timed up and go) AND (resistance exercise OR strength training OR power training OR weight lifting OR muscle strengthening)

**ANZCTR**

(Balance) AND (resistance exercise OR strength training OR weight lifting OR power training)

**WHO ICTRP**

(star excursion OR Y-test OR Y test OR Y-balance test OR Romberg test OR single-leg stance OR functional reach test OR limits of stability OR limits-of-stability OR timed up and go) AND (resistance exercise OR strength training OR power training OR weight lifting OR muscle strengthening)

**31.Ren_2020 Efficacy and safety of dexmedetomidine as an adjuvant to local wound infiltration anaesthesia: A meta-analysis with trial sequential analysis of 23 randomised controlled trials**

**CT.GOV**

(Dexmedetomidine OR MVP 1440 OR Precedex OR Dexmedetomidine Hydrochloride OR Hydrochloride OR Dexmedetomidine OR Dexmedetomidine ) AND (wound infusion OR wound infiltration OR incision infiltration OR incision infusion )

**EUDRACT**

(Dexmedetomidine OR MVP 1440 OR Precedex OR Dexmedetomidine Hydrochloride OR Hydrochloride OR Dexmedetomidine OR Dexmedetomidine ) AND (wound infusion OR wound infiltration OR incision infiltration OR incision infusion )

**ANZCTR**

(wound infusion OR incision infiltration) AND (Dexmedetomidine OR MVP 1440 OR Hydrochloride)

**WHO ICTRP**

(Dexmedetomidine OR MVP 1440 OR Precedex OR Dexmedetomidine Hydrochloride OR Hydrochloride OR Dexmedetomidine OR Dexmedetomidine ) AND (wound infusion OR wound infiltration OR incision infiltration OR incision infusion )

**32.Alyoubi_2020 Efficacy and safety of mirogabalin treatment in patients with diabetic peripheral neuropathic pain: A systematic review and meta-analysis of randomised controlled trials**

**CT.GOV**

(Diabetic Peripheral Neuropathic Pain OR Diabetic Peripheral Neuropathy OR Peripheral Neuropathy OR Neuropathic Pain) AND (Mirogabalin OR Mirogabalin besylate OR Tarlige)

**EUDRACT**

(Diabetic Peripheral Neuropathic Pain OR Diabetic Peripheral Neuropathy OR Peripheral Neuropathy OR Neuropathic Pain) AND (Mirogabalin OR Mirogabalin besylate OR Tarlige)

**ANZCTR**

(Diabetic Peripheral Neuropathic Pain) AND (Mirogabalin OR Tarlige)

**WHO ICTRP**

(Diabetic Peripheral Neuropathic Pain) AND (Mirogabalin OR Tarlige)

**33.Shi_2020 Efficacy of Adherence-Enhancing Interventions for Immunosuppressive Therapy in Solid Organ Transplant Recipients: A Systematic Review and Meta-Analysis Based on Randomized Controlled Trials**

**CT.GOV**

(Compliance OR adherence OR cooperation OR medication adherence OR patient compliance OR patient adherence)

AND:

(Organ Transplantation OR Heart Transplantation OR Heart-Lung Transplantation OR Kidney Transplantation OR Liver Transplantation OR Pancreas Transplantation OR Renal Transplantation OR Kidney Grafting OR Hepatic Transplantation OR Pancreas Grafting)

------------------------------------------------------------------------------------------------------------------------

(Liver Grafting OR Lung Grafting OR Heart Grafting OR Cardiac Transplantation OR Lung Transplantation)

**EUDRACT**

(Compliance OR Patient Adherence OR Patient Cooperation OR Patient Non-Compliance OR Non Compliance OR Adherence OR Nonadherence OR Non Adherence OR Medication Adherence OR Patient Compliance) AND (Organ Transplantation OR Heart Transplantation OR Heart-Lung Transplantation OR Kidney Transplantation OR Liver Transplantation OR Pancreas Transplantation OR Renal Transplantation OR Kidney Grafting OR Hepatic Transplantation OR Pancreas Grafting OR Liver Grafting OR Lung Grafting OR Heart Grafting OR Cardiac Transplantation OR Lung Transplantation )

**ANZCTR**

(Compliance OR Patient Adherence) AND (transplantation OR Graft)

**WHO ICTRP**

(Compliance OR Patient Adherence) AND (transplantation OR Graft)

**34.Chiu_2020 Efficacy of gabexate mesilate in preventing post endoscopic retrograde cholangiopancreatography pancreatitis: A meta-analysis of randomized clinical trials**

**CT.GOV**

(Endoscopic retrograde cholangiopancreatography OR ERCP OR pancreatitis) AND (FOY OR FOY007 OR gabexate mesylate OR gabexate methanesulfonate OR 4-(6-guanidinohexanoyloxy) benzoic acid ethyl ester methanesulfonate)

**EUDRACT**

(Endoscopic retrograde cholangiopancreatography OR ERCP OR pancreatitis) AND (FOY OR FOY007 OR gabexate mesylate OR gabexate methanesulfonate OR 4-(6-guanidinohexanoyloxy) benzoic acid ethyl ester methanesulfonate)

**ANZCTR**

(Endoscopic retrograde cholangiopancreatography OR ERCP OR pancreatitis) AND (gabexate OR FOY)

**WHO ICTRP**

(Endoscopic retrograde cholangiopancreatography OR ERCP OR pancreatitis)AND (gabexate OR FOY OR FOY007 OR gabexate mesylate OR gabexate methanesulfonate)

**35. Miranda_2020 Efficacy of ropivacaine versus bupivacaine in preventing peribulbar block failure: A meta-analysis**

**CT.GOV**

Peribulbar AND (ropivacaine AND bupivacaine)

**EUDRACT**

(ropivacaine AND bupivacaine) AND (Peribulbar)

**ANZCTR**

(ropivacaine AND bupivacaine) AND (Peribulbar)

**WHO ICTRP**

(ropivacaine AND bupivacaine) AND (Peribulbar)

**37.Ye_2020 Immediate vs conventional loading of mandibular overdentures: A comprehensive systematic review and meta-analysis of randomized controlled trials**

**CT.GOV**

(implant OR dental implant OR overdenture) AND (immediate loading OR loading)

**EUDRACT**

(implant OR dental implant OR overdenture) AND (immediate loading OR loading)

**ANZCTR**

(implant OR dental implant OR overdenture) AND (immediate loading OR loading)

**WHO ICTRP**

(implant OR dental implant OR overdenture) AND (immediate loading OR loading)

**38.Lagowska_2020 Improvement of glucose metabolism in pregnant women through probiotic supplementation depends on gestational diabetes status: meta-analysis**

**CT.GOV**

(pregnancy OR gestation OR pregnant OR gestational diabetes OR GDM) AND ((probiotics OR bacteria) AND (supplementation OR supplement))

**EUDRACT**

((probiotics OR bacteria) AND (supplementation OR supplement)) AND (pregnancy OR gestation OR pregnant OR gestational diabetes OR GDM).

**ANZCTR**

((probiotics OR bacteria) AND (supplement)) AND (pregnancy OR pregnant OR gestational diabetes)

**WHO ICTRP**

((probiotics OR bacteria) AND (supplement)) AND (pregnancy OR pregnant OR gestational diabetes)

**39. Leng_2020 Internet-Based Supportive Interventions for Family Caregivers of People With Dementia: Systematic Review and Meta-Analysis**

**CT.GOV**

((Dementia OR Alzheimer) AND (Caregivers OR spouses OR Adult children OR Family OR home nursing OR caring OR carer OR caretaker OR caregiver OR home)) AND (Internet OR Web OR computer OR technology OR online OR electronic OR digital OR platform OR E-learning OR E-advice OR mHealth OR eHealth OR ICT OR Network OR telemedicine OR telehealth OR telecommunication OR Application OR interface OR tablet OR APP)

**EUDRACT**

((Dementia OR Alzheimer) AND (Caregivers OR spouses OR Adult children OR Family OR home nursing OR caring OR carer OR caretaker OR caregiver OR home)) AND ((Internet OR Web OR computer OR technology OR online OR electronic OR digital OR platform OR E-learning OR E-advice OR mHealth OR eHealth OR ICT OR Network OR telemedicine OR telehealth OR telecommunication OR Application OR interface OR tablet OR ipad OR APP))

**ANZCTR**

((Dementia OR Alzheimer) AND (Caregivers OR caretaker)) AND (Internet OR Web)

((Dementia OR Alzheimer) AND (Caregivers OR caretaker)) AND (OR computer OR technology OR online)

((Dementia OR Alzheimer) AND (Caregivers OR caretaker)) AND (electronic OR digital OR platform)

**WHO ICTRP**

((Dementia OR Alzheimer) AND (Caregivers OR caretaker)) AND (electronic OR digital OR platform OR internet OR Web OR Technology OR online)

**41.Choi_2020 L-Carnitine's Effect on the Biomarkers of Metabolic Syndrome: A Systematic Review and Meta-Analysis of Randomized Controlled Trials**

**CT.GOV**

(carnitine OR L-carnitine OR L-carnitine tartrate) AND (metabolic syndrome OR syndrome X OR Metabolic Syndrome X OR Insulin Resistance Syndrome X OR Metabolic X Syndrome OR Reaven Syndrome X OR Metabolic Cardiovascular Syndrome)

**EUDRACT**

(carnitine OR L-carnitine OR L-carnitine tartrate) AND (metabolic syndrome OR syndrome X OR Metabolic Syndrome X OR Insulin Resistance Syndrome X OR Metabolic X Syndrome OR Reaven Syndrome X OR Metabolic Cardiovascular Syndrome)

**ANZCTR**

(carnitine OR L-carnitine OR L-carnitine tartrate) AND (metabolic syndrome)

**WHO ICTRP**

(carnitine OR L-carnitine OR L-carnitine tartrate) AND (metabolic syndrome)

**42.Wu_2020 Meta-Analysis of Clinical Efficacy and Safety of Ligustrazine in the Treatment of Idiopathic Pulmonary Fibrosis**

**CT.GOV**

(Idiopathic pulmonary fibrosis OR Pulmonary fibrosisor OR Pulmonary interstitial fibrosis OR Interstitial lung disease OR IPF) AND (Ligustrazine OR Tetramethylpyrazine OR TMP OR Chuanxiongqin OR Chuanxiong rhizome OR Chuanxiong OR Ligusticum wallichii OR Ligustilide)

**EUDRACT**

(Idiopathic pulmonary fibrosis OR Pulmonary fibrosisor OR Pulmonary interstitial fibrosis OR Interstitial lung disease OR IPF) AND (Ligustrazine OR Tetramethylpyrazine OR TMP OR Chuanxiongqin OR Chuanxiong rhizome OR Chuanxiong OR Ligusticum wallichii OR Ligustilide)

**ANZCTR**

(Idiopathic pulmonary fibrosis OR Interstitial lung) AND (Ligustrazine OR Tetramethylpyrazine)

**WHO ICTRP**

(Idiopathic pulmonary fibrosis OR Interstitial lung) AND (Ligustrazine OR Tetramethylpyrazine OR TMP OR Chuanxiongqin OR Chuanxiong OR Ligusticum wallichii)

**44.Lin_2020 Negative pressure wound therapy for burn patients: A meta-analysis and systematic review NAME_2020: TITLE**

**CT.GOV**

(NPWT OR negative pressure) AND (burn)

**EUDRACT**

(NPWT OR negative pressure) AND (burn)

**ANZCTR**

(NPWT OR negative pressure) AND (burn)

**WHO ICTRP**

(NPWT OR negative pressure) AND (burn)

**45.Hamam_2020 Outcome comparison between wavefront-guided and wavefront-optimized photorefractive keratectomy: A systematic review and meta-analysis**

**CT.GOV**

(Myopia) AND (Wavefront‑guided photorefractive keratectomy OR Wavefront‑optimized photorefractive keratectomy)

**EUDRACT**

(Myopia) AND (Wavefront‑guided photorefractive OR Wavefront‑optimized photorefractive)

**ANZCTR**

(Myopia) AND (Wavefront‑guided photorefractive OR Wavefront‑optimized photorefractive)

**WHO ICTRP**

(Myopia) AND (Wavefront‑guided photorefractive OR Wavefront‑optimized photorefractive)

**47.Lex_2020 Perioperative Systemic Dexamethasone Reduces Length of Stay in Total Joint Arthroplasty: A Systematic Review and Meta-Analysis of Randomized Controlled Trials**

**CT.GOV**

(dexamethasone OR steroid OR corticosteroid OR glucocorticoid) AND (total hip arthroplasty OR hip arthroplasty OR total hip replacement OR hip replacement OR THA OR THR OR total knee arthroplasty OR knee arthroplasty OR total knee replacement OR knee replacement OR TKA OR TKR)

**EUDRACT**

(dexamethasone OR steroid OR corticosteroid OR glucocorticoid) AND (total hip arthroplasty OR hip arthroplasty OR total hip replacement OR hip replacement OR THA OR THR OR total knee arthroplasty OR knee arthroplasty OR total knee replacement OR knee replacement OR TKA OR TKR)

**ANZCTR**

(steroid OR corticosteroid) AND (hip arthroplasty OR hip replacement)

**WHO ICTRP**

(steroid OR corticosteroid) AND (hip arthroplasty OR hip replacement)

**49.Dudi-Venkata_2020 Safety and efficacy of laxatives after major abdominal surgery: systematic review and meta-analysis**

**CT.GOV**

(Laxatives OR Aperients OR Cathartic) AND (Abdominal surgery OR Digestive System Surgical Procedure OR Digestive surgery OR Colorectal surgery OR Colectomy OR General surgery OR Surgical oncology OR Gynaecologic surgery OR Laparoscopy OR Laparotomy OR Abdominal OR gastrointestinal OR colon)

**EUDRACT**

(Laxatives OR Aperients OR Cathartic) AND (Abdominal surgery OR Digestive System Surgical Procedure OR Digestive surgery OR Colorectal surgery OR Colectomy OR General surgery OR Surgical oncology OR Gynaecologic surgery OR Laparoscopy OR Laparotomy OR Abdominal OR gastrointestinal OR colon)

**ANZCTR**

(Laxatives OR Aperients OR Cathartic) AND (Surgery OR Laparoscopy)

(Laxatives OR Aperients OR Cathartic) AND (Surgical OR Laparotomy)

(Laxatives OR Aperients OR Cathartic) AND (Colectomy OR Abdominal)

(Laxatives OR Aperients OR Cathartic) AND (digestive OR Gynaecologic)

(Laxatives OR Aperients OR Cathartic) AND (oncology OR Colorectal)

**WHO ICTRP**

(Laxatives OR Aperients OR Cathartic) AND (Surgery OR Laparoscopy OR Surgical OR laparotomy OR colectomy OR abdominal OR digestive OR gynaecologic OR oncology OR colorectal)

**50.Hung_2020 Safety of two common laparoscopic inguinal herniorrhaphy approaches: an updated systematic review with meta-analysis of randomized clinical trials**

**CT.GOV**

(Total extra peritoneal OR total extraperitoneal OR tep) AND (TAPP OR trans-abdominal pre-peritoneal OR transabdominal preperitoneal)

**EUDRACT**

(Total extra peritoneal OR total extraperitoneal OR tep) AND (TAPP OR trans-abdominal pre-peritoneal OR transabdominal preperitoneal)

**ANZCTR**

(Hernia) AND ( total extra peritoneal OR total extraperitoneal)

**WHO ICTRP**

(Hernia) AND ( total extra peritoneal OR total extraperitoneal)

**51.Zaman_2020 Single-port laparoscopic appendicectomy versus conventional three-port approach for acute appendicitis in children: a systematic review and meta-analysis**

**CT.GOV**

(Appendectomy OR Appendicectomy) AND(Laparoscopic OR Single-port OR Three-ports OR Conventional)

**EUDRACT**

(Appendectomy OR Appendicectomy) AND (Laparoscopic OR Single-port OR Three-ports OR Conventional)

**ANZCTR**

(Appendectomy OR Appendicectomy) AND (Laparoscopic OR Single-port OR Three-ports OR Conventional)

**WHO ICTRP**

(Appendectomy OR Appendicectomy) AND (Laparoscopic OR Single-port OR Three-ports OR Conventional)

**52.Zou_2020 Sodium-glucose cotransporter inhibitors as add-on therapy in addition to insulin for type 1 diabetes mellitus: A meta-analysis of randomized controlled trials**

**CT.GOV**

(Diabetes Mellitus, Type 1 OR type 1 diabetes OR type 1 diabetes mellitus OR T1DM OR Diabetes Mellitus, Insulin-Dependent OR Diabetes Mellitus, Insulin Dependent OR Insulin-Dependent Diabetes Mellitus) AND (Sodium-Glucose Transporter 1 OR Sodium-Glucose Transporter 2 OR Sodium-Glucose Transport Proteins OR Sodium-glucose cotransporter OR SGLT OR SGLT1 OR SGLT2 OR Dapagliflozin OR Canagliflozin OR Empagliflozin OR Ertugliflozin OR Ipragliflozin)

**EUDRACT**

(type 1 diabetes OR type 1 diabetes mellitus OR Insulin dependent) AND (Sodium-Glucose Transporter OR Dapagliflozin OR Canagliflozin OR SGLT1 OR SGLT2 OR Empagliflozin OR Ipragliflozin OR Luseogliflozin OR Tofogliflozin OR Sotagliflozin OR Gliflozins)

**ANZCTR**

(type 1 diabetes OR Insulin dependent) AND (Canagliflozin OR SGLT1 OR SGLT2 OR Empagliflozin)

(type 1 diabetes OR Insulin dependent) AND (Ipragliflozin OR Luseogliflozin)

(type 1 diabetes OR Insulin dependent) AND ( Tofogliflozin OR Sotagliflozin OR Gliflozins)

**WHO ICTRP**

(type 1 diabetes OR type 1 diabetes mellitus OR T1DM OR Insulin-Dependent Diabetes Mellitus) AND (Sodium-Glucose Transporter 1 OR Sodium-Glucose Transporter 2 OR Sodium-Glucose Transport Proteins

OR Sodium-glucose cotransporter OR SGLT OR SGLT1 OR SGLT2 OR Dapagliflozin OR Canagliflozin OR Empagliflozin OR Ertugliflozin OR Ipragliflozin OR Luseogliflozin OR Tofogliflozin OR Sotagliflozin OR Gliflozins)

**53.Munoz-Viguera Speech and language therapy treatment on hypokinetic dysarthria in Parkinson disease: Systematic review and meta-analysis**

**CT.GOV**

(Parkinson disease OR Parkinson OR PD) AND (Hypokinetic Dysarthria OR dysarthria) AND (Speech therapy OR Rehabilitation of Speech and Language Disorders OR Voice Training OR LSVT OR Lee Silverman Voice Treatment)

**EUDRACT**

((Parkinson disease OR Parkinson OR PD) AND (Hypokinetic Dysarthria OR dysarthria)) AND (Speech therapy OR Rehabilitation of Speech and Language Disorders OR Voice Training OR LSVT OR Lee Silverman Voice Treatment)

**ANZCTR**

(Parkinsons AND Dysarthria) AND (Speech therapy OR Rehabilitation of Speech)

(Parkinsons AND Dysarthria) AND (Language Disorders OR Voice Training)

(Parkinsons AND Dysarthria) AND (LSVT OR Lee Silverman Voice Treatment)

**WHO ICTRP**

(Parkinson OR Parkinsons) AND (Speech therapy OR Rehabilitation of Speech and Language Disorders OR Voice Training OR LSVT OR Lee Silverman Voice Treatment)

**54.Yu_2020 Systematic review and meta-analysis of randomized controlled trials assessing the impact of Baduanjin exercise on cognition and memory in patients with mild cognitive impairment**

**CT.GOV**

(Ba Duan Jin OR Baduanjin OR Eight Section Brocades OR Eight-Section Brocade OR Eight Segments of Brocade) AND

(Cognitive Dysfunction OR Cognition Disorders OR Dementia OR Amnesia OR Cognition OR Memory Disorders OR Alzheimer Disease OR Executive Function OR Attention OR Lewy Body Disease OR Huntington Disease OR neurocognit OR dementia)

**EUDRACT**

(Ba Duan Jin OR Baduanjin OR Eight Section Brocades OR Eight-Section Brocade OR Eight Segments of Brocade) AND (Cognitive Dysfunction OR Cognition Disorders OR Dementia OR Amnesia OR Cognition OR Memory Disorders OR Alzheimer Disease OR Executive Function OR Attention OR Lewy Body Disease OR Huntington Disease OR neurocognit OR dementia)

(Ba Duan Jin OR Baduanjin OR Eight Section Brocades OR Eight-Section Brocade OR Eight Segments of Brocade) AND (amnesia OR alzheimer OR executive function OR attention OR memory OR MCI OR CI OR MMSE OR NCSE OR MoCA OR CDT OR WAIS OR ADAS-Cog OR VADAS-Cog OR SIB OR WHO-UCLA AVLT OR FAB OR Stroop)

**ANZCTR**

(Ba Duan Jin OR Baduanjin) AND (Cognitive Dysfunction OR Dementia)

(Eight Section Brocades OR Eight-Section Brocade OR Eight Segments of Brocade) AND Dementia

(Ba Duan Jin OR Baduanjin) AND Alzheimer

**WHO ICTRP**

(Ba Duan Jin OR Baduanjin OR Eight Section Brocades OR Eight-Section Brocade OR Eight Segments of Brocade) AND (Cognitive Dysfunction OR Cognition Disorders OR Dementia OR Amnesia OR Cognition OR Memory Disorders OR Alzheimer Disease OR Executive Function OR Attention OR Lewy Body Disease OR Huntington Disease OR neurocognit OR dementia)

**55.Langa_2020 The effect of cetylpyridinium chloride mouthrinse as adjunct to toothbrushing compared to placebo on interproximal plaque and gingival inflammation-a systematic review with meta-analyses**

**CT.GOV**

(Gingivitis OR gingival inflammation ) AND (Cetylpyridinium OR CPC OR 1-hexadecylpyridinium chloride OR acetoquat OR ammonyx CPC OR ceepryn chloride OR cepacol OR plax OR cetamium OR dobendan OR ipanol OR Merothol OR pristacin OR pyrisept OR asept OR mouthrinse OR mouthwash )

**EUDRACT**

(Gingivitis OR gingival inflammation ) AND (Cetylpyridinium OR CPC OR 1-hexadecylpyridinium chloride OR acetoquat OR ammonyx CPC OR ceepryn chloride OR cepacol OR plax OR cetamium OR dobendan OR ipanol OR Merothol OR pristacin OR pyrisept OR asept OR mouthrinse OR mouthwash )

**ANZCTR**

(Gingivitis OR gingival inflammation ) AND (Cetylpyridinium)

**WHO ICTRP**

(Gingivitis OR gingival inflammation ) AND (Cetylpyridinium OR CPC OR 1-hexadecylpyridinium chloride OR acetoquat OR ammonyx CPC OR ceepryn chloride OR cepacol OR plax OR cetamium OR dobendan OR ipanol OR Merothol OR pristacin OR pyrisept OR asept OR mouthrinse OR mouthwash )

**56.Shahnifar_2020 The effect of chromium supplementation on apolipoproteins: A systematic review and meta-analysis of randomized clinical trials**

**CT.GOV**

(Chromium) AND (Hyperlipidemias OR Hypercholesterolemia OR Hyperlipidemic OR Dyslipidemia OR Dyslipidemic OR Hypercholesterolaemia OR Hypercholesterolemic OR Hypercholesterolaemic OR Dyslipidemias OR Hypercholesterolemia)

**EUDRACT**

Chromium AND (Hyperlipidemias OR Hypercholesterolemia OR Hyperlipidemic OR Dyslipidemia OR Dyslipidemic OR Hypercholesterolaemia OR Hypercholesterolemic OR Hypercholesterolaemic OR Dyslipidemias OR Hypercholesterolemia)

**ANZCTR**

Chromium AND (Hyperlipidemias OR Hypercholesterolemia)

**WHO ICTRP**

Chromium AND (Hyperlipidemias OR Hypercholesterolemia OR Hyperlipidemic OR Dyslipidemia OR Dyslipidemic OR Hypercholesterolaemia OR Hypercholesterolemic OR Hypercholesterolaemic OR Dyslipidemias OR Hypercholesterolemia)

**57.Heshmati_2021 The effect of cinnamon supplementation on glycemic control in women with polycystic ovary syndrome: A systematic review and meta-analysis**

**CT.GOV**

(Polycystic Ovary Syndrome OR Ovary Syndrome OR Polycystic Ovary OR Polycystic ovary disease OR Stein- Leventhal Syndrome OR Stein Leventhal Syndrome OR Sclerocystic Ovarian Degeneration) AND

(Cinnamon OR Cinnamomum zeylanicum OR Cinnamomum OR true cinnamon OR Ceylon cinnamon)

**EUDRACT**

(Polycystic Ovary Syndrome OR Ovary Syndrome OR Polycystic Ovary OR Polycystic ovary disease OR Stein- Leventhal Syndrome OR Stein Leventhal Syndrome OR Sclerocystic Ovarian Degeneration) AND (Cinnamon OR Cinnamomum zeylanicum OR Cinnamomum OR true cinnamon OR Ceylon cinnamon)

**ANZCTR**

Cinnamon AND Polycystic Ovary syndrome

**WHO ICTRP**

(polycystic ovary OR polycystic ) AND (Cinnamon OR Cinnamomum zeylanicum OR Cinnamomum OR true cinnamon OR Ceylon cinnamon)

**58.Jamali 2020 The effect of cinnamon supplementation on lipid profiles in patients with type 2 diabetes: A systematic review and meta-analysis of clinical trials**

**CT.GOV**

(Cinnamon OR Cinnamom) AND (Diabetes OR T2DM OR type 2 diabetes mellitus OR diabetes mellitus OR type 2 diabetes OR Diabetic OR Diabet)

**EUDRACT**

(Cinnamon OR Cinnamom) AND (Diabetes OR T2DM OR type 2 diabetes mellitus OR diabetes mellitus OR type 2 diabetes)

**ANZCTR**

(Cinnamon OR Cinnamom) AND (Diabetes OR T2DM OR type 2 diabetes mellitus OR diabetes mellitus)

**WHO ICTRP**

(Cinnamon OR Cinnamom) AND (Diabetes OR T2DM OR type 2 diabetes mellitus OR diabetes mellitus OR type 2 diabetes OR Diabetic OR Diabet)

**59.Fu_2020 The Effect of Perioperative Music on Medication Requirement and Hospital Length of Stay: A Meta-analysis**

**CT.GOV**

(Music OR music therapy OR (musical OR musicotherap^*^)) AND :

(Surgical Procedures, Operative OR postoperative complications OR Anesthesiology OR perioperative nursing OR Operating Rooms OR recovery room OR Surgery)

**-------------**

(preoperative OR perioperative OR Postoperative OR Operative OR Intraoperative OR interoperative OR Anesthesia OR Anaesthesia OR perianesthesia OR peranesthesia OR Perianesthetic OR peranaesthetic OR Preanesthesia OR Postanesthesia OR postanaesthesia)

**EUDRACT**

(Music OR music therapy OR musical OR musicotherapy) AND (Surgical Procedures, Operative OR postoperative complications OR Anesthesiology OR perioperative nursing OR Operating Rooms OR recovery room OR Surgery)

**ANZCTR**

(Music OR music therapy OR musical OR musicotherapy) AND (Anaesthesia OR Surgery OR Operation)

**WHO ICTRP**

(Music OR music therapy OR musical OR musicotherapy) AND (Surgical Procedures, Operative OR postoperative complications OR Anesthesiology OR perioperative nursing OR Operating Rooms OR recovery room OR Surgery)

**60. Clark_2020 The effect of psyllium supplementation on blood pressure: a systematic review and meta-analysis of randomized controlled trials**

**CT.GOV**

(psyllium OR mucilage OR lunelax OR Metamucil OR ispaghul OR plantago OR iso­gel OR reguval) AND (Blood Pressure OR Hypertension OR Pre­hypertension OR SBP OR DBP)

**EUDRACT**

(psyllium OR mucilage OR lunelax OR Metamucil OR ispaghul OR plantago OR iso­gel OR reguval) AND (Blood Pressure OR Hypertension OR Pre­hypertension OR SBP OR DBP)

**ANZCTR**

(psyllium) AND (Blood Pressure OR Hypertension OR Pre­hypertension OR SBP OR DBP)

**WHO ICTRP**

(psyllium) AND (Blood Pressure OR Hypertension OR Pre­hypertension OR SBP OR DBP)

**61.Yang_2020 The effect of Tai Ji and Qigong in patients with chronic obstructive pulmonary disease: A systematic review and meta-analyses**

**CT.GOV**

(chronic obstructive pulmonary disease) AND(Tai Ji OR Qigong OR Ba Duan Jin OR Yi Jin Jing OR Wu Qin Xi OR Liu Zi Jue OR Daoyin yangsheng gong OR Shierduanjin OR Mawangdui guidance OR Big dance OR Tai Ji stick)

**EUDRACT**

(chronic obstructive pulmonary disease) AND ( Tai Ji OR Qigong OR Ba Duan Jin OR Yi Jin Jing OR Wu Qin Xi OR Liu Zi Jue OR Daoyin yangsheng gong OR Shierduanjin OR Mawangdui guidance OR Big dance OR Tai Ji stick)

**ANZCTR**

(chronic obstructive pulmonary disease) AND ( Tai Ji OR Qigong OR Ba Duan Jin OR Yi Jin Jing)

(chronic obstructive pulmonary disease) AND (Wu Qin Xi OR Liu Zi Jue OR Daoyin yangsheng gong)

**WHO ICTRP**

(chronic obstructive pulmonary disease) AND ( Tai Ji OR Qigong OR Ba Duan Jin OR Yi Jin Jing OR Wu Qin Xi OR Liu Zi Jue OR Daoyin yangsheng gong OR Shierduanjin OR Mawangdui guidance OR Big dance OR Tai Ji stick)

**66.Guillem_2020 The Effects of Resistance Training on Blood Pressure in Preadolescents and Adolescents: A Systematic Review and Meta-Analysis**

**CT.GOV**

((hypertension OR blood pressure) AND (children OR preadolescents OR youth))

AND

(resistance training OR weight training OR strength training)

**EUDRACT**

(hypertension AND children) AND (resistance training OR weight training OR strength training)

((hypertension OR blood pressure) AND (children OR preadolescents OR youth)) AND (resistance training OR weight training OR strength training)

**ANZCTR**

((hypertension OR blood pressure) AND (children OR preadolescents OR youth)) AND (training)

**WHO ICTRP**

(Hypertension OR blood pressure) AND (children OR preadolescents OR youth) AND (resistance training OR weight training OR strength training)

**67.Zhu_2020 The Efficacy and Safety of Combined Adductor Canal Block with Periarticular Anesthetic Injection Following Primary Total Knee Arthroplasty: A Meta-Analysis**

**CT.GOV**

(total knee arthroplasty OR total knee replacement) AND (adductor canal block OR saphenous nerve block OR peripheral nerve block OR periarticular anesthetic injection OR periarticular infiltration OR local infiltration analgesia)

**EUDRACT**

(total knee arthroplasty OR total knee replacement) AND (adductor canal block)

(total knee arthroplasty OR total knee replacement) AND (adductor canal block AND periarticular)

**ANZCTR**

(total knee arthroplasty OR total knee replacement) AND (adductor canal block AND periarticular)

**WHO ICTRP**

(total knee arthroplasty OR total knee replacement) AND (adductor canal block OR saphenous nerve block OR peripheral nerve block OR periarticular anesthetic injection OR periarticular infiltration OR local infiltration analgesia)

**71.Shen_2020 THE IMPACT OF POST-RADIOTHERAPY EXERCISE ON WOMEN WITH BREAST CANCER: A META-ANALYSIS OF RANDOMIZED CONTROLLED TRIALS**

**CT.GOV**

(Breast cancer OR breast neoplasms) AND (radiotherapy) AND (Exercise OR resistance training OR yoga)

**EUDRACT**

(Breast cancer AND radiotherapy) AND (Exercise OR resistance training OR yoga)

**ANZCTR**

(Breast cancer AND radiotherapy) AND (Exercise OR resistance training OR yoga)

**WHO ICTRP**

(Breast cancer OR breast neoplasms) AND (radiotherapy) AND (Exercise OR resistance training OR yoga)

**73.Yang_2020 The optimal immune checkpoint inhibitors combined with chemotherapy for advanced non-small-cell lung cancer: a systematic review and meta-analysis**

**CT.GOV**

(Non small cell lung carcinoma OR lung carcinoma OR non-small cell OR non-small cell lung cancer)

AND (drug therapies OR Drug therapy OR Chemotherapy OR Pharmacotherapy OR chemotherapies) AND ( Nivolumab OR ONO-4538 OR MDX-1106 OR BMS-936558 OR MK-3475 OR MDX-1106 OR ONO-4538 OR Keytruda OR lambrolizumab OR pembrolizumab OR RG7446 OR antiPD-L1 OR atezolizumab OR Nivo OR SCH-900475 OR Tecentriq OR Opdivo)

**EUDRACT**

(Non small cell lung cancer OR non small cell lung carcinoma) AND (chemotherapy OR drug therapy OR pharmacotherapy) AND ( Nivolumab OR ONO-4538 OR MDX-1106 OR BMS-936558 OR MK-3475 OR MDX-1106 OR ONO-4538 OR Keytruda OR lambrolizumab OR pembrolizumab OR RG7446 OR antiPD-L1 OR atezolizumab OR Nivo OR SCH-900475 OR Tecentriq OR Opdivo)

**ANZCTR**

(Non small cell lung) AND (chemotherapy) AND ( Nivolumab OR ONO-4538 OR MDX-1106)

(Non small cell lung) AND (chemotherapy) AND (BMS-936558 OR MK-3475 OR MDX-1106)

(Non small cell lung) AND (chemotherapy) AND (Keytruda OR lambrolizumab )

(Non small cell lung cancer OR non small cell lung carcinoma) AND (chemotherapy)

(Non small cell lung cancer OR non small cell lung carcinoma) AND chemotherapy AND Nivolumab

(Non small cell lung cancer OR non small cell lung carcinoma) AND chemotherapy AND pembrolizumab

(Non small cell lung cancer OR non small cell lung carcinoma) AND chemotherapy AND atezolizumab

(Non small cell lung cancer OR non small cell lung carcinoma) AND chemotherapy AND lambrolizumab

(Non small cell lung cancer OR non small cell lung carcinoma) AND chemotherapy AND Keytruda

(Non small cell lung cancer OR non small cell lung carcinoma) AND chemotherapy AND antiPD-L1

**WHO ICTRP**

(Non small cell lung carcinoma OR lung carcinoma OR non-small cell OR non-small cell lung cancer) AND (drug therapies OR Drug therapy OR Chemotherapy OR Pharmacotherapy OR chemotherapies) AND ( Nivolumab OR ONO-4538 OR MDX-1106 OR BMS-936558 OR MK-3475 OR MDX-1106 OR ONO-4538 OR Keytruda OR lambrolizumab OR pembrolizumab OR RG7446 OR antiPD-L1 OR atezolizumab OR Nivo OR SCH-900475 OR Tecentriq OR Opdivo)

**74. Lin_2020 Therapeutic efficacy and safety of Kangfuxin in combination with rabeprazole in the treatment of peptic ulcer: A systematic review and meta-analysis**

**CT.GOV**

(rabeprazole AND Kangfuxin) AND

(Peptic Ulcer OR Gastroduodenal Ulcer OR Stomach Ulcer OR Gastric Ulcer OR Duodenal Ulcer OR Curling Ulcer)

**EUDRACT**

(rabeprazole AND Kangfuxin) AND (Peptic Ulcer OR Gastroduodenal Ulcer OR Stomach Ulcer OR Gastric Ulcer OR Duodenal Ulcer OR Curling Ulcer)

**ANZCTR**

(rabeprazole AND Kangfuxin) AND (Peptic Ulcer OR Gastroduodenal Ulcer OR Stomach Ulcer )

(rabeprazole AND Kangfuxin) AND (Gastric Ulcer OR Duodenal Ulcer OR Curling Ulcer)

**WHO ICTRP**

(rabeprazole AND Kangfuxin) AND (Peptic Ulcer OR Gastroduodenal Ulcer OR Stomach Ulcer OR Gastric Ulcer OR Duodenal Ulcer OR Curling Ulcer)

**75.Hoshijima_2020 Use of the GlideScope does not lower the hemodynamic response to tracheal intubation more than the Macintosh laryngoscope: a systematic review and meta-analysis**

**CT.GOV**

(GlideScope OR Macintosh) AND (operation OR emergent OR urgent)

**EUDRACT**

(GlideScope OR Macintosh) AND (operation OR emergent OR urgent)

**ANZCTR**

(GlideScope OR Macintosh) AND (operation OR emergent OR urgent)

**WHO ICTRP**

(GlideScope OR Macintosh) AND (operation OR emergent OR urgent)

**76.Yates_2020 Venous thromboembolism risk with JAK inhibitors: A Meta-analysis**

**CT.GOV**

(Tofacitinib OR Baricitinib OR Upadacitinib OR Filgotinib) AND

(Rheumatoid OR Psoriatic arthritis OR Ankylosing spondylitis OR Axial spondyloarthritis OR Ulcerative colitis OR Crohns)

**EUDRACT**

(Tofacitinib OR Baricitinib OR Upadacitinib OR Filgotinib) AND (Rheumatoid OR Psoriatic arthritis OR Ankylosing spondylitis OR Axial spondyloarthritis OR Ulcerative colitis OR Crohns)

**ANZCTR**

(Tofacitinib OR Baricitinib OR Upadacitinib OR Filgotinib) AND (Rheumatoid OR Psoriatic arthritis)

(Tofacitinib OR Baricitinib OR Upadacitinib OR Filgotinib) AND (Axial spondyloarthritis)

(Tofacitinib OR Baricitinib OR Upadacitinib OR Filgotinib) AND (Ulcerative colitis OR Crohns)

**WHO ICTRP**

(Tofacitinib OR Baricitinib OR Upadacitinib OR Filgotinib) AND (Rheumatoid OR Psoriatic arthritis OR Ankylosing spondylitis OR Axial spondyloarthritis OR Ulcerative colitis OR Crohns)

**77.Zhou_2020 Yiqi Yangyin Huoxue Method in Treating IdiopathicPulmonary Fibrosis: A Systematic Review and Meta-Analysis of Randomized Controlled Trials**

**CT.GOV**

(idiopathic pulmonary Fibrosis OR pulmonary Fibrosis OR pulmonary interstitial Fibrosis OR idiopathic pulmonary interstitial Fibrosis OR IPF OR Feibi OR Feiwei ) AND:

(Yiqi Yangyin OR Yiqi Huoxue OR Yiqi Yangyin Huoxue OR tonifying qi and nourishing Yin OR supplementing qi and promoting blood OR nourishing Yin and promoting blood)

----------------------------------------------------------------------------------------------------------------------------

(Traditional Chinese medicine OR Chinese medicinal herb OR Chinese herbal medicine OR combination of Chinese traditional and western medicine OR TCM OR CHM)

**EUDRACT**

(idiopathic pulmonary Fibrosis OR pulmonary Fibrosis OR pulmonary interstitial Fibrosis OR idiopathic pulmonary interstitial Fibrosis OR IPF OR Feibi OR Feiwei ) AND (Yiqi Yangyin OR Yiqi Huoxue OR Yiqi Yangyin Huoxue OR tonifying qi and nourishing Yin OR supplementing qi and promoting blood OR nourishing Yin and promoting blood OR Traditional Chinese medicine OR Chinese medicinal herb OR Chinese herbal medicine OR combination of Chinese traditional and western medicine OR TCM OR CHM)

**ANZCTR**

(pulmonary Fibrosis OR Feibi OR Feiwei) AND (Yiqi Yangyin OR Yiqi Huoxue OR Yiqi Yangyin Huoxue)

**WHO ICTRP**

(idiopathic pulmonary Fibrosis OR pulmonary Fibrosis OR pulmonary interstitial Fibrosis OR idiopathic pulmonary interstitial Fibrosis OR IPF OR Feibi OR Feiwei ) AND (Yiqi Yangyin OR Yiqi Huoxue OR Yiqi Yangyin Huoxue OR tonifying qi and nourishing Yin OR supplementing qi and promoting blood OR nourishing Yin and promoting blood OR Traditional Chinese medicine OR Chinese medicinal herb OR Chinese herbal medicine OR combination of Chinese traditional and western medicine OR TCM OR CHM)

**78.D’Elia_2020 100% Fruit juice intake and cardiovascular risk: a systematic review and meta-analysis of prospective and randomised controlled studies**

**CT.GOV**

(Fruit juice) AND (Cardiovascular OR cerebrovascular OR stroke OR coronary heart OR blood pressure OR hypertension OR lipid OR cholesterol OR triglyceride OR HDL OR LDL OR glucose OR glycemia OR insulin OR HOMA OR weight OR BMI OR waist OR diabetes OR carotid)

(Fruit juice) AND (flow mediated dilation OR pulse wave velocity OR arterial compliance OR arterial)

**EUDRACT**

(Fruit juice) AND (Cardiovascular OR cerebrovascular OR stroke OR coronary heart OR blood pressure OR hypertension OR lipid OR cholesterol OR triglyceride OR HDL OR LDL OR glucose OR glycemia OR insulin OR HOMA OR weight OR BMI OR waist OR diabetes OR carotid)

(Fruit juice) AND (flow mediated dilation OR pulse wave velocity OR arterial compliance OR arterial)

**ANZCTR**

(Fruit juice) AND (Cardiovascular OR cerebrovascular OR stroke OR coronary heart OR blood pressure)

(Fruit juice) AND (hypertension OR lipid OR cholesterol OR triglyceride OR HDL OR LDL OR glucose)

(Fruit juice) AND (glycemia OR insulin OR HOMA OR weight OR BMI OR waist OR diabetes OR carotid)

(Fruit juice) AND (flow mediated dilation OR pulse wave velocity OR arterial compliance OR arterial)

**WHO ICTRP**

(Fruit juice) AND (Cardiovascular OR cerebrovascular OR stroke OR coronary heart OR blood pressure OR hypertension OR lipid OR cholesterol OR triglyceride OR HDL OR LDL OR glucose OR glycemia OR insulin OR HOMA OR weight OR BMI OR waist OR diabetes OR carotid)

(Fruit juice) AND (flow mediated dilation OR pulse wave velocity OR arterial compliance OR arterial)

**79.Limirio_2020 A clinical comparison of 1-piece versus 2-piece implants: A systematic review and meta-analysis**

**CT.GOV**

(Dental implants AND (one piece and two piece)

**EUDRACT**

(Dental implants AND (one piece and two piece)

**ANZCTR**

(Dental implants AND (one piece and two piece)

**WHO ICTRP**

(Dental implants AND (one piece and two piece)

**81.O’Shea_2020 Access to and sustainability of abortion services: a systematic review and meta-analysis for the National Institute of Health and Care Excellence-new clinical guidelines for England**

**CT.GOV**

(Abortion OR pregnancy termination OR abortion applicants OR aborted fetus OR Fetus death) AND (health services accessibility OR health care access OR health care utilization OR Health care delivery OR integrated health care system OR quality of health care OR Service use OR services OR infrastructure)

**EUDRACT**

(Abortion OR pregnancy termination OR abortion applicants OR aborted fetus OR Fetus death) AND (health services accessibility OR health care access OR health care utilization OR Health care delivery OR integrated health care system OR quality of health care OR Service use OR services OR infrastructure)

**ANZCTR**

(Abortion OR pregnancy termination) AND (health services accessibility OR infrastructure OR Quality)

(Abortion OR pregnancy termination) AND (health care access OR health care utilization )

(Abortion OR pregnancy termination) AND (Health care delivery OR services)

(Abortion OR pregnancy termination) AND (integrated health care system)

**WHO ICTRP**

(Abortion OR pregnancy termination OR abortion applicants OR aborted fetus OR Fetus death) AND (health services accessibility OR health care access OR health care utilization OR Health care delivery OR integrated health care system OR quality of health care OR Service use OR services OR infrastructure)

**83.Reimert_2020 Are hip movement precautions effective in preventing prosthesis dislocation post hip arthroplasty using a posterior surgical approach? A systematic review and meta-analysis**

**CT.GOV**

(Hip arthroplasty OR Hip replacement OR total hip prosthesis OR hip joint OR hip hemiarthroplasty OR hemiarthroplasty OR Total joint OR hip OR THA OR THR) AND

(Precaution OR restriction OR occupational therapy education OR physical therapy education OR education OR preoperative education OR allied health education OR physician assistant education OR advice OR protocol OR instruction)

**EUDRACT**

(Hip arthroplasty OR Hip replacement OR total hip prosthesis OR hip joint OR hip hemiarthroplasty OR hemiarthroplasty OR Total joint OR hip OR THA OR THR) AND (Precaution OR restriction OR occupational therapy education OR physical therapy education OR education OR preoperative education OR allied health education OR physician assistant education OR advice OR protocol OR instruction)

**ANZCTR**

(Hip OR total joint) AND (OR allied health education OR physician assistant education OR advice)

(Hip OR total joint) AND ( protocol OR instruction OR Precaution OR restriction)

(Hip OR total joint) AND (occupational therapy education OR physical therapy education)

(Hip OR total joint) (education OR preoperative education)

**WHO ICTRP**

(Hip arthroplasty OR Hip replacement OR total hip prosthesis OR hip joint OR hip hemiarthroplasty OR hemiarthroplasty OR Total joint OR hip OR THA OR THR) AND (Precaution OR restriction OR occupational therapy education OR physical therapy education OR education OR preoperative education OR allied health education OR physician assistant education OR advice OR protocol OR instruction)

**86.Buneviciene_2020 Can mHealth interventions improve quality of life of cancer patients? A systematic review and meta-analysis**

**CT.GOV**

(Mobile health OR mobile application OR mobile app OR mobile health app OR mhealth) AND (Cancer)

**EUDRACT**

(Mobile health OR mobile application OR mobile app OR mobile health app OR mhealth) AND (Cancer)

**ANZCTR**

(Mobile health OR mobile application OR mobile app OR mobile health app OR mhealth) AND (Cancer)

**WHO ICTRP**

(Mobile health OR mobile application OR mobile app OR mobile health app OR mhealth) AND (Cancer)

**87.Onggo_2020 Comparable dislocation and revision rates for patients undergoing total hip arthroplasty with subsequent or prior lumbar spinal fusion: a meta-analysis and systematic review**

**CT.GOV**

(Total hip arthroplasty OR THA OR Total hip replacement OR THR) AND (Spinal fusion OR Spinal arthrodesis)

**EUDRACT**

(Total hip arthroplasty OR THA OR Total hip replacement OR THR) AND (Spinal fusion OR Spinal arthrodesis)

**ANZCTR**

(Total hip arthroplasty OR THA OR Total hip OR THR) AND (Spinal fusion OR Spinal arthrodesis)

**WHO ICTRP**

(Total hip arthroplasty OR THA OR Total hip replacement OR THR) AND (Spinal fusion OR Spinal arthrodesis)

**88.Dave_2020 Comparative Effectiveness of Entecavir Versus Tenofovir for Preventing Hepatocellular Carcinoma in Patients with Chronic Hepatitis B: A Systematic Review and Meta-Analysis**

**CT.GOV**

(hepatitis B) AND (Entecavir vs Tenofovir)

**EUDRACT**

(hepatitis B) AND (Entecavir vs Tenofovir)

**ANZCTR**

(hepatitis B) AND (Entecavir vs Tenofovir)

**WHO ICTRP**

(hepatitis B) AND (Entecavir vs Tenofovir)

**89.Liu_2020 Comparison of clinical outcomes with proximal femoral nail anti-rotation versus InterTAN nail for intertrochanteric femoral fractures: a meta-analysis**

**CT.GOV**

(hip OR femoral OR femoral intertrochanteric fracture OR intertrochanteric fracture OR trochanteric fractures OR intertrochanteric fractures OR extracapsular hip fractures OR extracapsular femoral fractures) AND:

((Intramedullary OR Internal) AND (Fracture Fixation)) AND (InterTAN nail OR InterTAN OR Integrated 2 screw derotation cephalomedullary device OR Integrated Lag Screws OR Intertrochanteric Antegrade Nail)

--------------

(PFNA OR Proximal femoral nail antirotation) AND (InterTAN nail OR InterTAN OR Integrated 2 screw derotation cephalomedullary device OR Integrated Lag Screws OR Intertrochanteric Antegrade Nail)

-----------

(single screw cephalomedullary nail OR PFNAII) AND (InterTAN nail OR InterTAN OR Integrated 2 screw derotation cephalomedullary device OR Integrated Lag Screws OR Intertrochanteric Antegrade Nail)

--------

(proximal femoral nail antirotation-Asia OR Helical Blade) AND (InterTAN nail OR InterTAN OR Integrated 2 screw derotation cephalomedullary device OR Integrated Lag Screws OR Intertrochanteric Antegrade Nail)

**EUDRACT**

((Intramedullary OR Internal) AND (Fracture Fixation)) AND (InterTAN nail OR InterTAN OR Integrated 2 screw derotation cephalomedullary device OR Integrated Lag Screws OR Intertrochanteric Antegrade Nail)

(PFNA OR Proximal femoral nail antirotation) AND (InterTAN nail OR InterTAN OR Integrated 2 screw derotation cephalomedullary device OR Integrated Lag Screws OR Intertrochanteric Antegrade Nail)

(single screw cephalomedullary nail OR PFNAII) AND (InterTAN nail OR InterTAN OR Integrated 2 screw derotation cephalomedullary device OR Integrated Lag Screws OR Intertrochanteric Antegrade Nail)

(proximal femoral nail antirotation-Asia OR Helical Blade) AND (InterTAN nail OR InterTAN OR Integrated 2 screw derotation cephalomedullary device OR Integrated Lag Screws OR Intertrochanteric Antegrade Nail)

**ANZCTR**

((Intramedullary OR Internal) AND (Fracture Fixation)) AND (InterTAN OR Integrated 2 screw )

(Intramedullary fracture OR Internal fracture) AND (Integrated Lag Screws OR Intertrochanteric)

(proximal femoral nail OR Helical Blade) AND (InterTAN nail OR integrated 2 screw)

(proximal femoral nail OR Helical Blade) AND (InterTAN OR integrated 2 screw)

**WHO ICTRP**

(hip fracture OR femoral fractures OR femoral intertrochanteric fracture OR intertrochanteric fracture OR trochanteric fractures OR intertrochanteric femur fracture OR intertrochanteric hip fractures OR extracapsular hip fractures OR extracapsular femoral fractures) AND (Intramedullary Fracture Fixation OR Internal Fracture Fixation OR PFNA OR Proximal femoral nail antirotation OR single screw cephalomedullary nail OR PFNAII OR proximal femoral nail antirotation-Asia OR Helical Blade) AND (InterTAN nail OR InterTAN OR Integrated 2 screw derotation cephalomedullary device OR Integrated Lag Screws OR Intertrochanteric Antegrade Nail)

**90. Zhou_2020 Comparison of Cranial Facet Joint Violation Rate and Four Other Clinical Indexes Between Robot-assisted and Freehand Pedicle Screw Placement in Spine Surgery: A Meta-analysis**

**CT.GOV**

**(**Robotics OR robot OR robotic) AND (spine OR Zygapophyseal Joint OR Pedicle Screws OR Zygapophyseal Joints OR Facet Joint OR Facet Joints OR Pedicle Screw)

**EUDRACT**

(Robotics OR robot OR robotic) AND (spine OR Zygapophyseal) (Robotics OR robot OR robotic) AND (spine OR Zygapophyseal Joint OR Pedicle Screws OR Zygapophyseal Joints OR Facet Joint OR Facet Joints OR Pedicle Screw)

**ANZCTR**

(Robotics OR robot OR robotic) AND (spine OR Zygapophyseal )

(Robotics OR robot OR robotic) AND ( Pedicle Screws OR Facet Joint )

**WHO ICTRP**

(Robotics OR robot OR robotic) AND (spine OR Zygapophyseal Joint OR Pedicle Screws OR Zygapophyseal Joints OR Facet Joint OR Facet Joints OR Pedicle Screw)

**92.Gottlieb_2021 Comparison of the Loop Technique With Incision and Drainage for Skin and Soft Tissue Abscesses: A Systematic Review and Meta-analysis**

**CT.GOV**

(Loop OR loops OR looped OR looping OR subcutaneous drain OR subcutaneous drains OR subcutaneous drainage OR abscess OR abscesses) AND (minimally invasive NOT brain abscess NOT brain abscesses NOT loop colostomy)

**EUDRACT**

(Loop OR loops OR looped OR looping OR subcutaneous drain OR subcutaneous drains OR subcutaneous drainage OR abscess OR abscesses) AND (minimally invasive NOT brain abscess NOT brain abscesses NOT loop colostomy)

**ANZCTR**

(Loop OR loops OR looped OR looping OR subcutaneous drain OR Abscess)

(Loop OR loops OR looped OR looping OR subcutaneous drain OR Abscess) AND (minimally invasive)

**WHO ICTRP**

(Loop OR loops OR looped OR looping OR subcutaneous drain OR subcutaneous drains OR subcutaneous drainage OR abscess OR abscesses) AND (minimally invasive NOT brain abscess NOT brain abscesses NOT loop colostomy)

**93.Seo_2020 Dangguijagyag-san for primary dysmenorrhea A PRISMA-compliant systematic review and meta-analysis of randomized-controlled trials**

**CT.GOV**

(Primary dysmenorrhea OR dysmenorrhea OR menstrual disorder OR menstruation disorders OR menstrual pain OR painful period OR cramps)

AND:

(Oriental medicine OR medicinal herb OR herbal medicine OR Plant Extracts OR herbal OR Herbal formula OR TCM OR decoction OR Chinese medicine OR Kampo medicine OR Kampo OR Traditional Korean medicine OR Traditional medicine)

OR (Dangguijagyag-san OR Danggwijagyaksan OR Dangguijagyagsan OR Tokishakuyakusan OR Tokisyakuyakusanryo OR TJ-23 OR Dang gui shao yao san)

**EUDRACT**

(Primary dysmenorrhea OR dysmenorrhoea OR menstrual disorder OR menstruation disorders OR menstrual pain OR painful period OR cramps) AND (Oriental medicine OR medicinal herb OR herbal medicine OR Plant Extracts OR herbal OR Herbal formula OR TCM OR decoction OR Chinese medicine OR Kampo medicine OR Kampo OR Traditional Korean medicine OR Traditional medicine OR Dangguijagyag-san OR Danggwijagyaksan OR Dangguijagyagsan OR Tokishakuyakusan OR Tokisyakuyakusanryo OR TJ-23 OR Dang gui shao yao san)

**ANZCTR**

(dysmenorrhea OR dysmenorrhoea) AND ( Chinese medicine OR Dangguijagyag-san OR Dangguijagyagsan)

(dysmenorrhea OR dysmenorrhoea) AND ( Kampo OR Tokishakuyakusan -san OR Dang gui shao yao san)

**WHO ICTRP**

(Primary dysmenorrhea OR dysmenorrhoea OR menstrual disorder OR menstruation disorders OR menstrual pain OR painful period OR cramps) AND (Oriental medicine OR medicinal herb OR herbal medicine OR Plant Extracts OR herbal OR Herbal formula OR TCM OR decoction OR Chinese medicine OR Kampo medicine OR Kampo OR Traditional Korean medicine OR Traditional medicine OR Dangguijagyag-san OR Danggwijagyaksan OR Dangguijagyagsan OR Tokishakuyakusan OR Tokisyakuyakusanryo OR TJ-23 OR Dang gui shao yao san)

**95.Luca_2020 Does the presence of parents in the dental operatory room influence children's behavior, anxiety and fear during their dental treatment? A systematic review**

**CT.GOV**

(Parents OR parent" OR parental OR mother OR father) AND (child OR preschool OR pediatric OR paediatric) AND (dental OR dentistry) AND (Visit OR visits OR treatment OR treatments OR restoration OR restorations OR Extraction OR Extractions OR Prophylaxis)

**EUDRACT**

(Parents OR parent OR parental OR mother OR father) AND (child OR preschool OR pediatric OR paediatric) AND (dental OR dentistry) AND (Visit OR visits OR treatment OR treatments OR restoration OR restorations OR Extraction OR Extractions OR Prophylaxis)

**ANZCTR**

(Parents) AND (child OR paediatric) AND (dental OR dentistry) AND (Visit OR treatment OR restoration)

(Parents) AND (child OR paediatric) AND (dental OR dentistry) AND (Extraction OR Prophylaxis)

(mother OR father) AND (child OR paediatric) AND (dental OR dentistry) AND (Visit OR treatment)

**WHO ICTRP**

(Parents OR parent OR parental OR mother OR father) AND (child OR preschool OR pediatric OR paediatric) AND (dental OR dentistry) AND (Visit OR visits OR treatment OR treatments OR restoration OR restorations OR Extraction OR Extractions OR Prophylaxis)

**96.See_2020 Effectiveness and Safety of Direct Oral Anticoagulants in an Asian Population with Atrial Fibrillation Undergoing Dialysis: A Population-Based Cohort Study and Meta-Analysis**

**CT.GOV**

(apixaban OR dabigatran OR rivaroxaban OR edoxaban) AND (warfarin)

AND

(atrial fibrillation) AND (dialysis OR end stage renal disease OR end stage kidney disease)

**EUDRACT**

(apixaban OR dabigatran OR rivaroxaban OR edoxaban) AND (warfarin) AND (atrial fibrillation) AND (dialysis OR end stage renal disease OR end stage kidney disease)

**ANZCTR**

(apixaban OR dabigatran OR rivaroxaban OR edoxaban) AND (warfarin) AND (atrial fibrillation)

(apixaban OR dabigatran OR rivaroxaban OR edoxaban) AND warfarin AND (dialysis OR end stage renal)

(apixaban OR dabigatran OR rivaroxaban OR edoxaban) AND warfarin AND end stage

(atrial fibrillation) AND (Renal OR Kidney) AND (Warfarin) AND (apixaban OR dabigatran)

(atrial fibrillation) AND (Renal OR Kidney) AND (Warfarin) AND (rivaroxaban OR edoxaban)

**WHO ICTRP**

apixaban OR dabigatran OR rivaroxaban OR edoxaban) AND (warfarin) AND (atrial fibrillation) AND (dialysis OR end stage renal disease OR end stage kidney disease)

**97.Mo_2020Effectiveness and safety of pneumococcal vaccines used alone or combined with influenza vaccination in dialysis patients: A systematic review and meta-analysis**

**CT.GOV**

(Pneumococcal Pneumonia OR Pneumonia OR Pneumovax OR Pneumonia OR bronchopneumonia OR Bronchial Pneumonia) AND (vaccines OR vaccination OR immunity OR immunization OR immune OR Pneumococcal Vaccines)

AND

(Chronic Kidney Failure OR Chronic Renal Insufficiency OR renal insufficiency OR kidney insufficiency OR renal impairment OR kidney impairment OR kidney dysfunction OR renal dysfunction OR dialysis OR hemodialysis OR Haemodialysis OR hematodialysis)

**EUDRACT**

(Pneumococcal Vaccines) AND (Chronic Kidney Failure OR Chronic Renal Insufficiency OR renal insufficiency OR kidney insufficiency OR renal impairment OR kidney impairment OR kidney dysfunction OR renal dysfunction OR dialysis OR hemodialysis OR Haemodialysis OR hematodialysis)

**ANZCTR**

(Pneumococcal OR pneumonia) AND (kidney OR Renal OR dialysis OR hemodialysis OR Haemodialysis)

(Pneumococcal OR pneumonia) AND (hematodialysis)

**WHO ICTRP**

((Pneumococcal Pneumonia OR Pneumonia OR Pneumovax OR Pneumonia OR bronchopneumonia OR Bronchial Pneumonia) AND (vaccines OR vaccination OR immunity OR immunization OR immune OR Pneumococcal Vaccines) ) AND (Chronic Kidney Failure OR Chronic Renal Insufficiency OR renal insufficiency OR kidney insufficiency OR renal impairment OR kidney impairment OR kidney dysfunction OR renal dysfunction OR dialysis OR hemodialysis OR Haemodialysis OR hematodialysis)

**99.Ojeda_2020 Effects of Beta-Alanine Supplementation on Physical Performance in Aerobic-Anaerobic** **Transition Zones: A Systematic Review and Meta-Analysis**

**CT.GOV**

(b-alanine OR beta-alanine OR b-alanine supplementation OR beta-alanine supplementation) AND (maximal aerobic speed OR maximal oxygen uptake OR maximal aerobic consumption OR endurance)

**EUDRACT**

(b-alanine OR beta-alanine OR b-alanine supplementation OR beta-alanine supplementation) AND (maximal aerobic speed OR maximal oxygen uptake OR maximal aerobic consumption OR endurance)

**ANZCTR**

(b-alanine OR beta-alanine) AND (aerobic OR oxygen uptake OR endurance)

**WHO ICTRP**

(b-alanine OR beta-alanine OR b-alanine supplementation OR beta-alanine supplementation) AND (maximal aerobic speed OR maximal oxygen uptake OR maximal aerobic consumption OR endurance)

**100.Ong_2021 Effects of psychological interventions on competitive anxiety in sport: A meta-analysis**

**CT.GOV**

(psychological OR mental skill) AND (experiment OR intervention) AND (athlete OR sport)

**EUDRACT**

(psychological OR mental skill) AND (experiment OR intervention) AND (athlete OR sport)

**ANZCTR**

(psychological OR mental skill) AND (experiment OR intervention) AND (athlete OR sport)

**WHO ICTRP**

(psychological OR mental skill) AND (experiment OR intervention) AND (athlete OR sport)

**101.Tleyjeh_2021 Efficacy and safety of tocilizumab in COVID-19 patients: a living systematic review and meta-analysis**

**CT.GOV**

(tocilizumab OR actemra* OR atizumab OR lusinex OR roactemra)

AND

(COVID-19 OR 19-ncov OR SARS-COV2 OR coronavirus19 OR coronavirus2019 OR SARS corona-virus 2)

**EUDRACT**

(tocilizumab OR actemra* OR atizumab OR lusinex OR roactemra) AND (COVID-19 OR 19-ncov OR SARS-COV2 OR coronavirus19 OR coronavirus2019 OR SARS corona-virus 2)

**ANZCTR**

(tocilizumab OR actemra* OR atizumab OR lusinex OR roactemra) AND (COVID-19 OR 19-ncov OR SARS-COV2)

(tocilizumab OR actemra* OR atizumab OR lusinex OR roactemra) AND (Corona)

**WHO ICTRP**

(tocilizumab OR actemra* OR atizumab OR lusinex OR roactemra) AND (COVID-19 OR 19-ncov OR SARS-COV2 OR coronavirus19 OR coronavirus2019 OR SARS corona-virus 2)

**102.Chu_2020 Efficacy and tolerability of theta-burst stimulation for major depression: A systematic** **review and meta-analysis**

**CT.GOV**

(Theta burst stimulation) AND (Major depressive disorder OR unipolar depression OR bipolar depression OR bipolar disorder)

**EUDRACT**

(Theta burst stimulation) AND (Major depressive disorder OR unipolar depression OR bipolar depression OR bipolar disorder)

**ANZCTR**

(Theta burst stimulation) AND (depressive OR Depression OR unipolar OR bipolar)

**WHO ICTRP**

(Theta burst stimulation) AND (Major depressive disorder OR unipolar depression OR bipolar depression OR bipolar disorder)

**103.Lima_2020 Elastic resistance training produces benefits similar to conventional resistance training in people with chronic obstructive pulmonary disease: Systematic review and meta-analysis**

**CT.GOV**

(COPD OR Chronic Obstructive Pulmonary Disease) AND (Resistance training OR elastic resistance OR elastic band OR elasticized band OR elastic)

**EUDRACT**

(COPD OR Chronic Obstructive Pulmonary Disease) AND (Resistance training OR elastic resistance OR elastic band OR elasticized band OR elastic)

**ANZCTR**

(COPD OR Chronic Obstructive Pulmonary) AND (Resistance OR elastic OR band)

**WHO ICTRP**

(COPD OR Chronic Obstructive Pulmonary Disease) AND (Resistance training OR elastic resistance OR elastic band OR elasticized band OR elastic)

**104.Baunwall_2020 Faecal microbiota transplantation for recurrent Clostridioides difficile infection: An updated systematic review and meta-analysis**

**CT.GOV**

(Fecal microbiota OR faecal microbiota OR stool transplant OR transplantation OR bacteriotherapy) AND

(CDI OR rCDI OR Clostridioides Difficile OR Clostridium Difficile OR Clostridium infection OR Recurrent Clostridium difficile infection)

**EUDRACT**

(Fecal microbiota OR faecal microbiota OR stool transplant OR transplantation OR bacteriotherapy) AND (CDI OR rCDI OR Clostridioides Difficile OR Clostridium Difficile OR Clostridium infection OR Recurrent Clostridium difficile infection)

**ANZCTR**

(faecal OR stool transplant OR transplantation OR bacteriotherapy) AND (CDI OR rCDI )

(faecal OR stool transplant OR transplantation OR bacteriotherapy) AND Clostridioides Difficile

(faecal OR stool transplant OR transplantation OR bacteriotherapy) AND (Clostridium Difficile)

(faecal OR stool transplant OR transplantation OR bacteriotherapy) AND (Clostridium infection)

**WHO ICTRP**

(Fecal microbiota OR faecal microbiota OR stool transplant OR transplantation OR bacteriotherapy) AND (CDI OR rCDI OR Clostridioides Difficile OR Clostridium Difficile OR Clostridium infection OR Recurrent Clostridium difficile infection)

**105.Zhou_2020 Folate intake, markers of folate status and oral clefts: An updated set of systematic reviews and meta-analyses**

**CT.GOV**

(Folic acid OR folate OR folacin vitamins OR dietary supplements OR Multivitamin OR dietary supplement OR food supplement OR neutraceutical) AND (Cleft lip OR cleft palate OR cleft OR harelip)

**EUDRACT**

(Folic acid OR folate OR folacin vitamins OR dietary supplements OR Multivitamin OR dietary supplement OR food supplement OR neutraceutical) AND (Cleft lip OR cleft palate OR cleft OR harelip)

**ANZCTR**

(Folic acid OR folate OR folacin) AND (Cleft lip OR cleft palate OR cleft OR harelip)

**WHO ICTRP**

(Folic acid OR folate OR folacin vitamins OR dietary supplements OR Multivitamin OR dietary supplement OR food supplement OR neutraceutical) AND (Cleft lip OR cleft palate OR cleft OR harelip)

**106.Huang_**2020 High-Flow Nasal Cannula in Hypercapnic Respiratory Failure: A Systematic Review and Meta-Analysis

**CT.GOV**

(HFNC OR high-flow nasal cannula OR high-flow oxygen therapy OR nasal high-flow oxygen therapy OR Nasal Highflow OR nasal high-flow OR High-Velocity Nasal Insufflation OR high flow oxygen OR long-term humidification) AND (Chronic Obstructive Pulmonary Disease OR COPD OR Chronic Obstructive Airway Disease OR Chronic Obstructive Lung Disease OR Chronic Airflow Obstruction OR hypercapnia OR hypercapnia OR hypercarbia OR CO2 retention)

**EUDRACT**

(HFNC OR high-flow nasal cannula OR high-flow oxygen therapy OR nasal high-flow oxygen therapy OR Nasal Highflow OR nasal high-flow OR High-Velocity Nasal Insufflation OR high flow oxygen OR long-term humidification) AND (Chronic Obstructive Pulmonary Disease OR COPD OR Chronic Obstructive Airway Disease OR Chronic Obstructive Lung Disease OR Chronic Airflow Obstruction OR hypercapnia OR hypercapnia OR hypercarbia OR CO2 retention)

(high-flow nasal cannula OR high-flow oxygen therapy) AND (Chronic Obstructive Pulmonary Disease OR COPD OR Chronic Obstructive Airway Disease OR Chronic Obstructive Lung Disease OR Chronic Airflow Obstruction OR hypercapnia OR hypercapnia OR hypercarbia OR CO2 retention)

**ANZCTR**

(HFNC OR high-flow OR high velocity) AND (COPD OR Chronic obstructive OR Hypercapnia OR Hypercarbia)

(HFNC OR high-flow OR high velocity) AND CO2 retention

**WHO ICTRP**

(HFNC OR high-flow nasal cannula OR high-flow oxygen therapy OR nasal high-flow oxygen therapy OR Nasal Highflow OR nasal high-flow OR High-Velocity Nasal Insufflation OR high flow oxygen OR long-term humidification) AND (Chronic Obstructive Pulmonary Disease OR COPD OR Chronic Obstructive Airway Disease OR Chronic Obstructive Lung Disease OR Chronic Airflow Obstruction OR hypercapnia OR hypercapnia OR hypercarbia OR CO2 retention)

**107.Kim_2020 Immune checkpoint inhibitor therapy may increase the incidence of treatment-related necrosis after stereotactic radiosurgery for brain metastases: a systematic review and meta-analysis**

**CT.GOV**

(Brain metastasis) AND (immunotherapy OR immune checkpoint inhibitor OR immune checkpoint blockade OR CTLA4 OR CTLA-4 OR PD1 OR PD-1 OR PD-L1 OR ipilimumab OR nivolumab OR pembrolizumab)

**EUDRACT**

(Brain metastasis) AND (immunotherapy OR immune checkpoint inhibitor OR immune checkpoint blockade OR CTLA4 OR CTLA-4 OR PD1 OR PD-1 OR PD-L1 OR ipilimumab OR nivolumab OR pembrolizumab)

**ANZCTR**

(Brain metastasis) AND (immunotherapy OR immune checkpoint OR OR CTLA4 OR CTLA-4 OR PD1 OR PD-1)

Brain metastasis) AND (PD-L1 OR ipilimumab OR nivolumab OR pembrolizumab)

**WHO ICTRP**

(Brain metastasis) AND (immunotherapy OR immune checkpoint inhibitor OR immune checkpoint blockade OR CTLA4 OR CTLA-4 OR PD1 OR PD-1 OR PD-L1 OR ipilimumab OR nivolumab OR pembrolizumab)

**109.Sandberg_2020 Ketamine for the treatment of prehospital acute pain: a systematic review of benefit and harm**

**CT.GOV**

(Ketamine) AND (emergency medical services OR Ambulances OR Ambulance OR Prehospital OR Pre-hospital OR out of hospital OR Paramed OR emergency medical technicians)

**EUDRACT**

(Ketamine) AND (emergency medical services OR Ambulances OR Ambulance OR Prehospital OR Pre-hospital OR out of hospital OR Paramed OR emergency medical technicians)

**ANZCTR**

Ketamine AND (emergency medical services OR Ambulances OR Ambulance OR Prehospital)

Ketamine AND (Pre-hospital OR out of hospital OR Paramed OR emergency medical technicians)

**WHO ICTRP**

(Ketamine) AND (emergency medical services OR Ambulances OR Ambulance OR Prehospital OR Pre-hospital OR out of hospital OR Paramed OR emergency medical technicians)

**110.Wu_2020 Laparoscopic sleeve gastrectomy versus Roux-en-Y gastric bypass for quality of life: a systematic review and meta-analysis**

**CT.GOV**

(Laparoscopic sleeve gastrectomy OR sleeve gastrectomy OR LSG OR SG) AND (laparoscopic Roux-en-Y gastric bypass OR gastric bypass OR LRYGB OR GB)

**EUDRACT**

(Laparoscopic sleeve gastrectomy OR sleeve gastrectomy OR LSG OR SG) AND (laparoscopic Roux-en-Y gastric bypass OR gastric bypass OR LRYGB OR GB)

**ANZCTR**

(Laparoscopic sleeve gastrectomy OR sleeve gastrectomy OR LSG OR SG) AND (laparoscopic Roux-en-Y)

**WHO ICTRP**

(Laparoscopic sleeve gastrectomy OR sleeve gastrectomy OR LSG OR SG) AND(gastric bypass OR LRYGB )

**111.Qiu_2020 Meta-regression analysis of the efficacy of alendronate for prevention of glucocorticoid-induced fractures**

**CT.GOV**

(Alendronate OR Bisphosphonate OR MK 217 OR MK217 OR MK-217 OR Aminohydroxybutane Bisphosphonate OR Fosamax)

AND (Glucocorticoids OR Steroid-Induced OR Steroid Induced OR Corticosteroid-Induced OR Corticosteroid Induced OR glucocorticoid users OR Steroids OR glucocorticoid OR steroid OR methylprednisolone OR prednisone OR prednisolone)

**EUDRACT**

(Alendronate OR Bisphosphonate OR MK 217 OR MK217 OR MK-217 OR Aminohydroxybutane Bisphosphonate OR Fosamax) AND (Glucocorticoids OR Steroid-Induced OR Steroid Induced OR Corticosteroid-Induced OR Corticosteroid Induced OR glucocorticoid users OR Steroids OR glucocorticoid MK 217 OR MK217 OR MK-217 OR Aminohydroxybutane Bisphosphonate OR Fosamax) AND (Glucocorticoids)OR steroid OR methylprednisolone OR prednisone OR prednisolone)

**ANZCTR**

(Alendronate OR Bisphosphonate) AND (Glucocorticoids OR Steroid Induced OR Corticosteroid Induced)

(Alendronate OR Bisphosphonate) AND( glucocorticoid OR steroid OR methylprednisolone OR prednisone )

(Alendronate OR Bisphosphonate) AND (prednisolone)

(MK 217 OR MK217 OR MK-217 OR Aminohydroxybutane Bisphosphonate OR Fosamax) AND (Glucocorticoids)

(MK 217 OR MK217 OR MK-217 OR Aminohydroxybutane Bisphosphonate OR Fosamax) AND (steroid)

**WHO ICTRP**

(Alendronate OR Bisphosphonate OR Fosamax) AND (Glucocorticoids OR Steroid Induced OR Corticosteroid Induced OR Steroid OR glucocorticoid OR prednisolone OR methylprednisolone)

**113.Rodriguez-Luna_2020 Outcomes of Laparoscopic Splenectomy for Treatment of Splenomegaly: A Systematic Review and Meta-analysis**

**CT.GOV**

(Laparoscopy OR minimal invasive OR hand assisted OR laparoscopic OR robotic surgical procedures OR

robot OR Vinci OR hand)

AND (splenectomy OR splenectomies OR splenomegalies OR splenomegaly OR splenomegaly)

(Splenectomy OR laparoscopic splenectomy OR robotic splenectomy OR Robotic splenectomy OR splenomegalies OR splenomegaly)

**EUDRACT**

(Splenectomy OR laparoscopic splenectomy OR robotic splenectomy OR Robotic splenectomy) AND (Splenomegaly OR splenomegalies)

(Laparoscopy OR minimal invasive OR hand assisted OR laparoscopic OR robotic surgical procedures OR robot OR Vinci OR hand) AND (splenectomy OR splenectomies OR splenomegalies OR splenomegaly OR splenomegaly)

**ANZCTR**

(Splenectomy OR laparoscopic splenectomy OR robotic splenectomy OR Robotic splenectomy)

**WHO ICTRP**

(Splenectomy OR laparoscopic splenectomy OR robotic splenectomy OR Robotic splenectomy) AND (Splenomegaly OR splenomegalies)

**114.Keerthana_2020 Performance comparison of vibration devices on orthodontic tooth movement - A systematic review and meta-analysis**

**CT.GOV**

(VIBRATION OR Acceledent OR Powered tooth brush) AND (orthodontics OR tooth movement OR distalization)

**EUDRACT**

(VIBRATION OR Acceledent OR Powered tooth brush) AND (orthodontics OR tooth movement OR distalization)

**ANZCTR**

(VIBRATION OR Acceledent OR Powered tooth brush) AND (orthodontics OR tooth movement)

(VIBRATION OR Acceledent OR Powered tooth brush) AND (distalization)

**WHO ICTRP**

(VIBRATION OR Acceledent OR Powered tooth brush) AND (orthodontics OR tooth movement OR distalization)

**116.Goldberg_2020 Post-acute psychological effects of classical serotonergic psychedelics: a systematic review and meta-analysis**

**CT.GOV**

(bipolar OR depression OR emotional OR mental OR psychiatric OR psychosis OR schizophrenia OR mood OR PTSD OR post-traumatic stress disorder OR ADHD OR attention deficit OR substance OR alcohol OR addiction OR eating OR anorexia OR bulimia OR binge) AND (psilocybin OR lysergic acid diethylamide OR LSD OR ayahuasca OR psychedelic)

**EUDRACT**

(bipolar OR depression OR emotional OR mental OR psychiatric OR psychosis OR schizophrenia OR mood OR PTSD OR post-traumatic stress disorder OR ADHD OR attention deficit OR substance OR alcohol OR addiction OR eating OR anorexia OR bulimia OR binge) AND (psilocybin OR lysergic acid diethylamide OR LSD OR ayahuasca OR psychedelic)

**ANZCTR**

(psilocybin OR lysergic acid diethylamide OR LSD OR ayahuasca OR psychedelic) AND (psychosis)

(psilocybin OR lysergic acid diethylamide OR LSD OR ayahuasca OR psychedelic) AND (Psychiatric)

(psilocybin OR lysergic acid diethylamide OR LSD OR ayahuasca OR psychedelic) AND (bipolar)

(psilocybin OR lysergic acid diethylamide OR LSD OR ayahuasca OR psychedelic) AND (mental)

(psilocybin OR lysergic acid diethylamide OR LSD OR ayahuasca OR psychedelic) AND (mood)

(psilocybin OR lysergic acid diethylamide OR LSD OR ayahuasca OR psychedelic) AND (eating)

(psilocybin OR lysergic acid diethylamide OR LSD OR ayahuasca OR psychedelic) AND (bulimia)

(psilocybin OR lysergic acid diethylamide OR LSD OR ayahuasca OR psychedelic) AND (ADHD)

(psilocybin OR lysergic acid diethylamide OR LSD OR ayahuasca OR psychedelic) AND (Post traumatic)

**WHO ICTRP**

(bipolar OR depression OR emotional OR mental OR psychiatric OR psychosis OR schizophrenia OR mood OR PTSD OR post-traumatic stress disorder OR ADHD OR attention deficit OR substance OR alcohol OR addiction OR eating OR anorexia OR bulimia OR binge) AND (psilocybin OR lysergic acid diethylamide OR LSD OR ayahuasca OR psychedelic)

**118.Liu_2020 Repeat hepatic resection VS radiofrequency ablation for the treatment of recurrent hepatocellular carcinoma: an updated meta-analysis**

**CT.GOV**

recurrent hepatocellular carcinoma OR intrahepatic recurrence OR recurrent liver cancer

AND

(radiofrequency ablation) AND (repeated hepatic resection OR re-hepatectomy OR repeat hepatectomy OR hepatic resection OR repeated resection OR reresection OR liver resection OR surgical resection OR redo hepatectomy OR repeat Liver Resection)

**EUDRACT**

(recurrent hepatocellular carcinoma OR intrahepatic recurrence OR recurrent liver cancer) AND (radiofrequency ablation) AND (repeated hepatic resection OR re-hepatectomy OR repeat hepatectomy OR hepatic resection OR repeated resection OR reresection OR liver resection OR surgical resection OR redo hepatectomy OR repeat Liver Resection)

(radiofrequency ablation) AND (repeated hepatic resection OR re-hepatectomy OR repeat hepatectomy OR hepatic resection OR repeated resection OR reresection OR liver resection OR surgical resection OR redo hepatectomy OR repeat Liver Resection)

**ANZCTR**

(hepatocellular carcinoma OR liver) AND (radiofrequency ablation) AND (repeated hepatic resection)

(Hepatic OR liver) AND (radiofrequency ablation) AND (liver resection OR surgical)

**WHO ICTRP**

(hepatocellular carcinoma OR intrahepatic recurrence OR recurrent liver cancer) AND (radiofrequency ablation) AND ( re-hepatectomy OR repeat hepatectomy OR hepatic resection OR repeated resection OR reresection OR liver resection OR surgical resection OR redo hepatectomy OR repeat Liver Resection)

**119.Veld_2020 Self-expandable metal stent (SEMS) placement or emergency surgery as palliative treatment for obstructive colorectal cancer: A systematic review and meta-analysis**

**CT.GOV**

(Colorectal Neoplasms OR Large intestine OR large bowel OR colon OR colorectal OR rectal OR rectum) AND (Stents OR stent)

**EUDRACT**

(Colorectal Neoplasms OR Large intestine OR large bowel OR colon OR colorectal OR rectal OR rectum) AND (Stents OR stent)

**ANZCTR**

(Colorectal Neoplasms OR Large intestine OR bowel OR colon OR colorectal OR rectal) AND (stent)

**WHO ICTRP**

(Colorectal Neoplasms OR Large intestine OR large bowel OR colon OR colorectal OR rectal OR rectum) AND (Stents OR stent)

**120.Anantha-Narayanan_2020 Systematic review and meta-analysis of outcomes of lower extremity peripheral arterial interventions in patients with and without chronic kidney disease or end-stage renal disease**

**CT.GOV**

(Peripheral arterial disease OR peripheral artery disease OR peripheral vascular ) AND (chronic renal insufficiency OR chronic kidney disease OR chronic kidney failure OR chronic renal failure OR end stage renal disease OR esrd OR haemodialysis) AND

(Endovascular OR Intervention) OR (bypass AND surgery) OR (Bypass AND transplants) OR (Bypass AND graft) OR transplantation OR grafting

**EUDRACT**

(Peripheral arterial disease OR peripheral artery disease OR peripheral vascular ) AND (chronic renal insufficiency OR chronic kidney disease OR chronic kidney failure OR chronic renal failure OR end stage renal disease OR esrd OR haemodialysis) AND (Endovascular OR Intervention) OR (bypass AND surgery) OR (Bypass AND transplants) OR (Bypass AND graft) OR transplantation OR grafting)

**ANZCTR**

(Peripheral arterial) AND (chronic renal) AND (endovascular OR bypass OR transplant OR graft)

(peripheral vascular) AND (renal OR kidney OR esrd OR haemodialysis) AND(Endovascular)

Peripheral vascular AND (renal OR kidney) AND (graft OR bypass OR transplantation)

**WHO ICTRP**

(Peripheral arterial disease OR peripheral artery disease OR peripheral vascular diseases) AND

((Endovascular OR Intervention) OR (bypass AND surgery) OR (Bypass AND transplants) OR (Bypass AND graft) OR transplantation OR grafting)

**121.Dohos_2020 Systematic review with meta-analysis: the effects of immunomodulator or biological withdrawal from mono- or combination therapy in inflammatory bowel disease**

**CT.GOV**

(Inflammatory Bowel Diseases OR IBD OR UC OR Ulcerative Colitis OR Crohn) AND:

(reduction OR deescalation OR withdraw OR discontinuation OR cessation OR stop OR interval widening) AND (Immunosuppressive agent OR antimetabolites OR immunomodulator OR methotrexate OR MTX OR azathioprine OR mercaptopurine OR 6MP)

----

(reduction OR deescalation OR withdraw OR discontinuation OR cessation OR stop OR interval widening) AND (thiopurine OR Infliximab OR Certolizumab Pegol OR Adalimumab OR vedolizumab OR ustekinumab OR golimumab OR IL-6 monoclonal)

----

(reduction OR deescalation OR withdraw OR discontinuation OR cessation OR stop OR interval widening) AND (Anti tnfalpha OR Antitnf OR antitumor necrosis OR anti tumor necrosis OR TNF OR anti tnf OR Tumor necrosis factor)

**EUDRACT**

(Inflammatory Bowel Diseases OR IBD OR UC OR Ulcerative Colitis OR Crohn) AND (reduction OR deescalation OR withdraw OR discontinuation OR cessation OR stop OR interval widening) AND (Immunosuppressive agent OR antimetabolites OR immunomodulator OR methotrexate OR MTX OR azathioprine OR mercaptopurine OR 6MP)

(reduction OR deescalation OR withdraw OR discontinuation OR cessation OR stop OR interval widening) AND (thiopurine OR Infliximab OR Certolizumab Pegol OR Adalimumab OR vedolizumab OR ustekinumab OR golimumab OR IL-6 monoclonal)

(reduction OR deescalation OR withdraw OR discontinuation OR cessation OR stop OR interval widening) AND (Anti tnfalpha OR Antitnf OR antitumor necrosis OR anti tumor necrosis OR TNF OR anti tnf OR Tumor necrosis factor)

**ANZCTR**

(Inflammatory bowel OR Ulcerative colitis) AND (withdraw OR de-escalation) AND (immunosuppressive)

(crohn OR Ulcerative colitis) AND (withdraw OR de-escalation) AND (methotrexate)

(crohn OR Ulcerative colitis) AND (withdraw OR de-escalation) AND (adalimumab)

(crohn OR Ulcerative colitis) AND (withdraw OR de-escalation) AND (tumour nectotic)

**WHO ICTRP**

(Inflammatory Bowel Diseases OR IBD OR UC OR Ulcerative Colitis OR Crohn) AND (reduction OR deescalation OR withdraw OR discontinuation OR cessation OR stop OR interval widening) AND (Immunosuppressive agent OR antimetabolites OR immunomodulator OR methotrexate OR MTX OR azathioprine OR mercaptopurine OR 6MP)

(reduction OR deescalation OR withdraw OR discontinuation OR cessation OR stop OR interval widening) AND (thiopurine OR Infliximab OR Certolizumab Pegol OR Adalimumab OR vedolizumab OR ustekinumab OR golimumab OR IL-6 monoclonal)

(reduction OR deescalation OR withdraw OR discontinuation OR cessation OR stop OR interval widening) AND (Anti tnfalpha OR Antitnf OR antitumor necrosis OR anti tumor necrosis OR TNF OR anti tnf OR Tumor necrosis factor)

**122.Beltsios_2020 Talc pleurodesis in malignant pleural effusion: a systematic review and meta-analysis**

**CT.GOV**

(Malignant pleural effusion OR pleural effusion) AND (talc AND pleurodesis)

**EUDRACT**

(Malignant pleural effusion OR pleural effusion) AND (talc AND pleurodesis)

**ANZCTR**

(Malignant pleural effusion OR pleural effusion) AND (talc AND pleurodesis)

**WHO ICTRP**

(Malignant pleural effusion OR pleural effusion) AND (talc AND pleurodesis)

**123.Yang_2020 The effect of donor human milk on the length of hospital stay in very low birthweight infants: a systematic review and meta-analysis**

**CT.GOV**

(donor milk OR donor breast milk OR banked milk OR pasteurized human milk OR donor human milk) AND (Very Low Birth Weight Infant)

**EUDRACT**

(donor milk OR donor breast milk OR banked milk OR pasteurized human milk OR donor human milk) AND (Very Low Birth Weight Infant)

**ANZCTR**

((donor milk OR donor breast milk) AND (Very Low Birth Weight Infant)

(banked milk OR pasteurized human milk OR donor human milk) AND (Very Low Birth Weight Infant)

**WHO ICTRP**

(donor milk OR donor breast milk OR banked milk OR pasteurized human milk OR donor human milk) AND (Very Low Birth Weight Infant)

**125.Mega_2020 The Outcome of Hydroxychloroquine in Patients Treated for COVID-19: Systematic Review and Meta-Analysis**

**CT.GOV**

(2019-ncov OR 2019 Novel corona virus OR COVID-19OR Coronavirus19)

AND

(Hydroxychloroquine OR Plaquenil OR Hydroxychloroquine sulphate)

**EUDRACT**

(2019-ncov OR 2019 Novel corona virus OR COVID-19OR Coronavirus19)

AND

(Hydroxychloroquine OR Plaquenil OR Hydroxychloroquine sulphate)

**ANZCTR**

(2019-ncov OR 2019 Novel corona virus ) AND (Hydroxychloroquine OR Plaquenil OR Hydroxychloroquine)

(COVID-19OR Coronavirus19) AND (Hydroxychloroquine OR Plaquenil OR Hydroxychloroquine)

**WHO ICTRP**

(2019-ncov OR 2019 Novel corona virus OR COVID-19OR Coronavirus19) AND (Hydroxychloroquine OR Plaquenil OR Hydroxychloroquine sulphate)

**126.Knisely_2020 The role of minimally invasive surgery in the care of women with ovarian cancer: A systematic review and meta-analysis**

**CT.GOV**

(Laparoscopic OR laparoscopy OR robot OR robotic OR minimally invasive) AND (ovarian cancer OR ovarian carcinoma OR ovary cancer OR ovary carcinoma)

**EUDRACT**

(Laparoscopic OR laparoscopy OR robot OR robotic OR minimally invasive) AND (ovarian cancer OR ovarian carcinoma OR ovary cancer OR ovary carcinoma)

**ANZCTR**

(Laparoscopic OR laparoscopy OR robot OR robotic OR minimally invasive) AND (ovarian cancer OR ovary)

**WHO ICTRP**

(Laparoscopic OR laparoscopy OR robot OR robotic OR minimally invasive) AND (ovarian cancer OR ovarian carcinoma OR ovary cancer OR ovary carcinoma)

**127.Prodromidis_2020 Timing of Anterior Cruciate Ligament Reconstruction and Relationship With Meniscal Tears: A Systematic Review and Meta-analysis**

**CT.GOV**

(Anterior cruciate ligament OR ACL) AND (Early OR delay OR Time) AND (surgery OR reconstruction OR reconstructive)

**EUDRACT**

(Anterior cruciate ligament OR ACL) AND (Early OR delay OR Time) AND (surgery OR reconstruction OR reconstructive)

**ANZCTR**

(Anterior cruciate ligament OR ACL) AND (Early OR delay OR Time) AND (surgery OR reconstruction)

**WHO ICTRP**

(Anterior cruciate ligament OR ACL) AND (Early OR delay OR Time) AND (surgery OR reconstruction OR reconstructive)

**128.Tien_2020 Transcranial direct current stimulation for improving ambulation after stroke: a systematic review and meta-analysis**

**CT.GOV**

(Transcranial direct current stimulation OR tDCS) AND (stroke)

**EUDRACT**

(Transcranial direct current stimulation OR tDCS) AND (stroke)

**ANZCTR**

(Transcranial direct current stimulation OR tDCS) AND (stroke)

**WHO ICTRP**

(Transcranial direct current stimulation OR tDCS) AND (stroke)

**129.Zhu_2020 Transversus Abdominis Plane Block With Liposomal Bupivacaine vs Regular Anesthetics for Pain Control After Surgery: A Systematic Review and Meta-Analysis**

**CT.GOV**

(Transverse abdominal block OR Transverse abdominal plane )

AND

(bupivacaine OR analgesia OR anesthesia OR liposomal bupivacaine) AND (block)

**EUDRACT**

(Transverse abdominal block OR Transverse abdominal plane )

AND

(bupivacaine OR analgesia OR anesthesia OR liposomal bupivacaine) AND (block)

**ANZCTR**

(Transverse abdominal) AND (bupivacaine OR analgesia OR liposomal bupivacaine) AND (block)

(Transverse abdominal) AND (Anaesthesia) AND (block)

**WHO ICTRP**

(Transverse abdominal block OR Transverse abdominal plane ) AND (bupivacaine OR analgesia OR anesthesia OR liposomal bupivacaine) AND (block)

**130.Hellegering_2020 Treatment of the extracranial carotid artery in tandem lesions during endovascular treatment of acute ischemic stroke: a systematic review and meta-analysis**

**CT.GOV**

(Stroke OR CVA OR cerebrovascular accident) OR (Carotid Arteries OR Carotid Stenosis OR carotid OR extracranial occlusion) AND (Tandem) AND

(Thrombolytic Therapy OR Mechanical Thrombolysis OR Intra-Arterial Infusions OR Endovascular Procedures OR intraarterial OR intra-arterial OR thrombolysis OR thrombolytic)

**EUDRACT**

(Stroke OR CVA OR cerebrovascular accident) OR (Carotid Arteries OR Carotid Stenosis OR carotid OR extracranial occlusion) AND (Tandem) AND

(Thrombolytic Therapy OR Mechanical Thrombolysis OR Intra-Arterial Infusions OR Endovascular Procedures OR intraarterial OR intra-arterial OR thrombolysis OR thrombolytic)

**ANZCTR**

(Stroke OR CVA OR Carotid OR extracranial occlusion) AND (Tandem) AND (Thrombolytic)

(Stroke OR CVA OR Carotid OR extracranial occlusion) AND (Tandem) AND (Endovascular)

(Stroke OR CVA OR Carotid OR extracranial occlusion) AND (Tandem) AND (procedures)

(Stroke OR CVA OR Carotid OR extracranial occlusion) AND (Tandem) AND ( intra arterial)

**WHO ICTRP**

(Stroke OR CVA OR cerebrovascular accident OR Carotid Arteries OR Carotid Stenosis OR carotid OR extracranial occlusion) AND (Tandem) AND (Thrombolytic Therapy OR Mechanical Thrombolysis OR Intra-Arterial Infusions OR Endovascular Procedures OR intraarterial OR intra-arterial OR thrombolysis OR thrombolytic)

**131.Hashimoto_2020 Use of antipyretics for preventing febrile seizure recurrence in children: a systematic review and meta-analysis**

**CT.GOV**

(Seizure) AND (febrile OR fever OR pyrexial OR convulsion)

AND (Antipyretic OR antifebrile OR acetaminophen OR paracetamol OR Ibuprofen )

**EUDRACT**

(Seizure) AND (febrile OR fever OR pyrexial OR convulsion)

AND (Antipyretic OR antifebrile OR acetaminophen OR paracetamol OR Ibuprofen )

**ANZCTR**

(Febrile seizure) AND (Antipyretic OR antifebrile OR acetaminophen OR paracetamol OR Ibuprofen)

(seizure) AND (Antipyretic OR antifebrile OR acetaminophen OR paracetamol OR Ibuprofen)

**WHO ICTRP**

(Seizure) AND (febrile OR fever OR pyrexial OR convulsion) AND (Antipyretic OR antifebrile OR acetaminophen OR paracetamol OR Ibuprofen)

## **Supplementary file 4. Impact of trial registers searches on summary estimates of meta-analyses**

**1. Yangoz 2020**

**Title of the systematic review:**

Comparison of the Effect of Educational and Self-Management Interventions on Adherence to Treatment in Hemodialysis Patients: A Systematic Review and Meta-Analysis of Randomized Controlled Trials

**Inclusion criteria:**

• Population: patients would be ≥18 years old and receiving hemodialysis treatment.

• Intervention: educational or self-management interventions.

• Comparison: studies that compared with routine care.

• Outcomes: studies in which adherence to treatment was evaluated.

• Study design: randomized controlled trial.

**Comparison assessed:**

Figure 2a. (The Change of IDWG on Adherence to Fluid Intake): Fluid Distribution Timetable (FDT) Group vs control

**Results:**


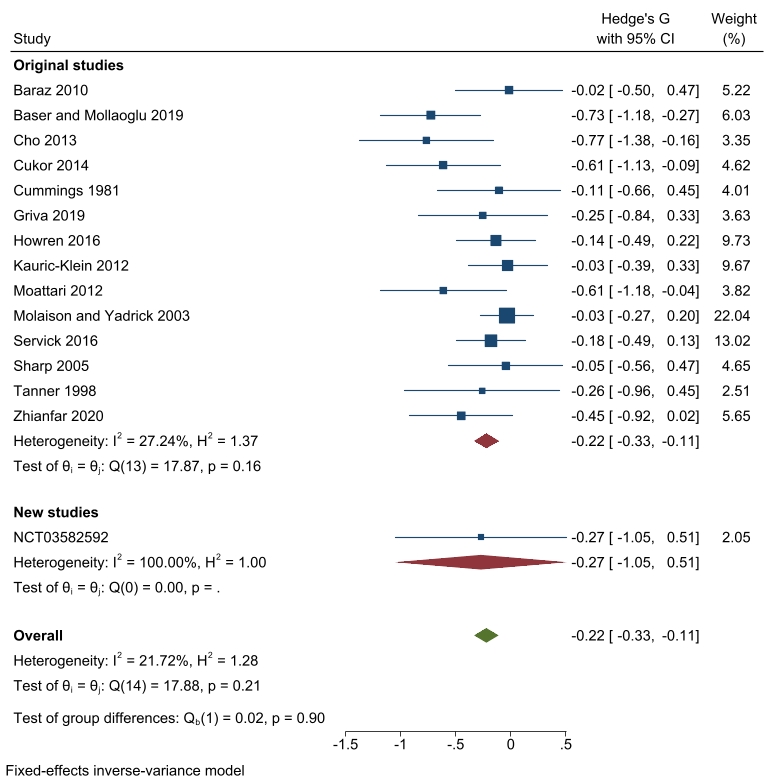


**2. Wood 2020**

**Title of the systematic review**

Dry cupping for musculoskeletal pain and range of motion: A systematic review and meta-analysis

**Inclusion criteria**

P: Participants were limited to adults (18 years and older) that received dry cupping treatment for musculoskeletal pain or restriction in range of motion. There were no restrictions to duration of pain period.

I: Treatment was limited to dry cupping therapy for a musculoskeletal condition as the sole intervention or combined with another modality.

C: Western medicine, sham cupping treatment or cross over intervention used as a control were included.

**Comparison assessed**

Figure 4. Effects of dry cupping vs. no treatment on pressure pain sensitivity for non-specific neck pain (Outcome measure: Pressure Pain Threshold).

**Results**

**3. Duarte-Garcia 2020**

**Title of the systematic review**

Effect of omega-3 fatty acids on systemic lupus erythematosus disease activity: A systematic review and meta-analysis

**Inclusion criteria**

The population of interest was adult patients (≥18 years) with SLE according to one of the accepted classification criteria.

All RCTs fulfilling the following criteria were included:

1. Trials compared omega-3 fatty acids supplementation to placebo or standard of care

2. SLE disease activity (before and after intervention or a difference in pre-and post-intervention) was reported

3. Duration of follow-up was at least 12 weeks.

**Comparison assessed**

Figure 2. Forest plot of the effects of Omega-3 fatty acids vs. placebo in trials

**Results**

**4. Parahiba 2020**

**Title of the systematic review**

Effect of testosterone supplementation on sarcopenic components in middle-aged and elderly men: A systematic review and meta-analysis

**Inclusion criteria**

randomized clinical trials of middle-aged men (age 40 to 60 years), elderly men (age > 60 years), or both (age > 40 years) that evaluated the effects of T supplementation on sarcopenic components (muscle mass, strength and physical performance).

Articles were required to mention at least one of the variables established to evaluate the outcomes, based on the consensus definition of sarcopenia (Cruz-Jentoft et al., 2019).

**Comparison assessed**

Figure 2a. Effect of testosterone supplementation compared to control or placebo on lean body mass (kg).

**Results**

**5. Navarro-Santana 2020**

**Title of the systematic review**

Effectiveness of Dry Needling for Myofascial Trigger Points Associated with Neck Pain Symptoms: An Updated Systematic Review and Meta-Analysis

**Inclusion criteria**

Population: Adults (older than 18 years) with TrPs in the cervical musculature associated with neck pain symptoms of musculoskeletal origin.

Intervention: Dry needling of muscle or tendon.

Comparator: Acceptable comparators were any sham or placebo dry needling, any control group without intervention, or any other type of physiotherapy intervention.

Interventions should be applied in isolation (self-stretching was permitted).

Outcomes: Pain intensity OR pain-related disability were considered as the primary outcomes.

Secondary outcomes included pressure pain thresholds OR cervical range of motion.

**Comparison assessed**

Figure 4. Mean differences (MD) comparing the short-term effects of dry needling alone against sham/placebo/waiting list/other forms of dry needling or manual therapy or other physical therapy.

**Results**

**6. Labata-Lezaun 2020**

**Title of the systematic review**

Effectiveness of Protein Supplementation Combined with Resistance Training on Muscle Strength and Physical Performance in Elderly: A Systematic Review and Meta-Analysis

**Inclusion criteria**

1. randomized controlled trial (RCT) study design,

2. adults aged >60,

3. healthy participants with or without sarcopenia condition,

4. intervention group with resistance training combined with protein supplementation,

5. comparison group with resistance training combined with placebo supplementation or no supplementation,

6. physical performance or strength as outcome, and

7. English language.

Comparison assessed

Figure 4. Impact of resistance training and protein supplement on lower limb strength. protein+ exercise vs placebo + exercise

**Results**

**7. Leng_2020**

**Title of the systematic review**

Internet-Based Supportive Interventions for Family Caregivers of People With Dementia: Systematic Review and Meta-Analysis

**Inclusion criteria**

1. participants were family caregivers who were currently providing caregiving support to people with dementia, defined as a family member such as their spouse or adult children providing unpaid care;

2. the intervention was a digital one delivered via any internet-based modality, which could include either single-component interventions or multiple-component interventions to family caregivers;

3. comparison was usual care or minimal support control by using paper materials, telephone, or email, etc;

4. primary outcomes included outcome variables related to family caregivers of people with dementia (depressive symptoms, caregiver burden, coping competence, perceived stress, caregiver reaction to

behavioral symptoms, anxiety, quality of life, and self-efficacy), and secondary outcomes included outcome variables related to people with dementia (care recipient’ quality of life and neuropsychiatric symptoms); and

5. to achieve high levels of evidence, we included only RCTs.

Comparison assessed

Figure 3. The effect of internet-based supportive interventions vs usual care on depressive symptoms.

**Results**

**8. Lex_2020**

**Title of the systematic review**

Perioperative Systemic Dexamethasone Reduces Length of Stay in Total Joint Arthroplasty: A Systematic Review and Meta-Analysis of Randomized Controlled Trials

**Inclusion criteria**

Only (RCTs) were included.

Population= patients undergoing Total Joint (hip/knee) Arthroplasty.

Studies evaluating the effect of any corticosteroid (dexamethasone, cortisone, hydrocortisone, methylprednisolone, betamethasone) administered.

systemically (either intravenous or oral) in any dose or timing regimen with follow-up until hospital discharge were included.

Patient outcomes: LOS, pain at rest or with activity, and PONV.

**Comparison assessed**

Figure 3a. Pain at rest comparing corticosteroid to placebo on postoperative day 0.

**Results**

**9. Dudi-Venkata_2020**

**Title of the systematic review**

Safety and efficacy of laxatives after major abdominal surgery: systematic review and meta-analysis

**Inclusion criteria**

RCTs conducted in patients aged more than 16 years undergoing elective open or minimally invasive major abdominal surgery.

Outcomes: specifically assessed the effect of laxatives on the return of gastrointestinal function, defined by time to passage of stool or using a validated measure such as GI-2 or GI-3. GI-2 is a composite measure of tolerance to solid diet for 24 h (no vomiting) and passage of stool, whereas GI-3 is a composite measure of tolerance to solid diet for 24 h (no vomiting) and passage of flatus26.

Eligible articles with a description of interventions directed towards stimulation of bowel motility, prevention of POI or reducing its duration, or facilitating the return of gastrointestinal function after surgery were included in the final analysis.

All gastrointestinal (colorectal, gastric, small bowel, hepatic, pancreatic resection), urological (nephrectomy, cystectomy, prostatectomy) and gynaecological (uterus and ovary resection, pelvic floor reconstruction) operations, undertaken for any indication, were considered as major abdominal surgery.

**Comparison assessed**

Figure 2a. Forest plots comparing time to passage of stool, laxatives vs no laxatives

**Results**

**10. Langa_2020**

**Title of the systematic review**

The effect of cetylpyridinium chloride mouthrinse as adjunct to toothbrushing compared to placebo on interproximal plaque and gingival inflammation-a systematic review with meta-analyses

**Inclusion criteria**

Randomized clinical trials;

Studies that involved adults at least 18 years old;

Individuals with any periodontal diagnosis were considered (higher level of dental plaque only, gingivitis or patients with periodontitis after periodontal therapy);

In the test group, mechanical hygiene in addition to CPC mouthwash;

In the control group, mechanical hygiene with placebo solution or mechanical hygiene alone;

The study had to evaluate any plaque or gingival index in the interproximal surfaces;

A minimum of 6 weeks of follow-up. Mouthwash had to be used continuously during the follow-up period.

**Comparison assessed**

Figure 3. Forest plot for the meta-analysis of the interproximal plaque index, CPC vs control groups

**Results**

**11. Yang_2020**

**Title of the systematic review**

The effect of Tai Ji and Qigong in patients with chronic obstructive pulmonary disease: A systematic review and meta-analyses

**Inclusion criteria**

1. study subjects were patients with an objective diagnosis of COPD according to pulmonary function (forced expiratory volume in first second/forced vital capacity (FEV1/FVC%) < 0.7) or the Global Initiative for COPD [25];

2. the intervention was defined as Chinese traditional exercise such as AJQ;

3. at least one of the following outcomes were reported: exercise capacity (6-minute walk distance (6MWD)), quality of life (COPD Assessment Test (CAT), the St. George's Respiratory Questionnaire (SGRQ)), pulmonary function (FEV1/FVC%, FEV1, predicted forced expiratory volume in 1 s(predicted FEV 1%));

4. randomized controlled trials (RCTs) were included;

5. papers published in the English and Chinese languages were included.

**Comparison assessed**

Figure 2. Forest plot of 6-minute walk distance, experimental vs control groups

**Results**

**12. Guillem_2020**

**Title of the systematic review**

The Effects of Resistance Training on Blood Pressure in Preadolescents and Adolescents: A Systematic Review and Meta-Analysis

**Inclusion criteria**

1. participants were youth (6–18 years);

2. the type of study was RCT,

3. at least one group had to perform RT and

Outcomes= SBP, DBP, BMI.

**Comparison assessed**

Figure 3. The effect of Resistance Training on systolic blood pressure (mmHg). Resistance training vs control

**Results**

**13.D’Elia_2020**

**Title of the systematic review**

100% Fruit juice intake and cardiovascular risk: a systematic review and meta-analysis of prospective and randomised controlled studies

**Inclusion criteria**

1. Original articles,

2. randomized controlled trials (RCTs),

3. studies involving the adult population,

4. studies in which there is an indication of the difference in outcomes—among which CV risk factors (e.g., blood pressure, lipid profile, glucose homeostasis, body weight) or CV damage (e.g., arterial stiffness, intima media thickness, flow-mediated dilation) between the intake of 100% FJ [24] and of control drink in one or more patient cohorts;

5. studies in which there are indications of the number of participants included in the exposed and control groups;

6. studies in which the length of intervention is at least 7 days.

**Comparison assessed**

Figure 3a. Forest plot of the effect of 100% fruit juice intake on systolic blood pressure (BP)

**Results**

**14. Buneviciene_2020**

**Title of the systematic review**

Can mHealth interventions improve quality of life of cancer patients? A systematic review and meta-analysis

**Inclusion criteria**

Patients with established cancer or cancer survivors.

Original research papers performed in humans and with their full texts available in English were considered for the review.

Randomized controls trials (RCT) and observational studies were both considered for inclusion.

Outcome= health-related quality of life.

**Comparison assessed**

Figure 3. pooled standardized mean difference comparing the change in HRQoL in mHealth vs. control

**Results**

**15. Keerthana_2020**

**Title of the systematic review**

Performance comparison of vibration devices on orthodontic tooth movement - A systematic review and meta-analysis

**Inclusion criteria**

P= Any age group of patients requiring fixed orthodontic treatment.

I= Vibrations using a powered tooth brush or Acceledent.

C= Orthodontic treatment without any intervention with vibrating devices

O= Any alteration in tooth movement.

Orthodontic tooth movement outcomes measured on study models or digitized models or intra oral scans or directly from patient’s mouth.

D= RCTs, Split mouth studies, Clinically controlled trials

**Comparison assessed**

Figure 3. Vibrations using a powered tooth brush or Acceledent vs Orthodontic treatment without any intervention with vibrating devices.

**Results**

**16. Hao_2020**

**Title of the systematic review**

A meta-analysis of efficacy of topical steroids in eosinophilic esophagitis: From the perspective of histologic, clinical, and endoscopic outcome

**Inclusion criteria**

1. RCTs comparing topical steroids with placebo in EoE;

2. histological, clinical and endoscopic improvement were reported;

3. the response rate could be extracted;

4. references from eligible articles or reviews were also assessed.

**Comparison assessed**

Figure 2a. Efficacy of topical steroids versus placebo for complete response.

**Results**

**17. Salah_2020**

**Title of the systematic review**

Effect of sodium-glucose cotransporter 2 inhibitors on cardiovascular and kidney outcomes-Systematic review and meta-analysis of randomized placebo-controlled trials

**Inclusion criteria**

1. randomized clinical trials (RCTs);

2. trials comparing an SGLT2i (i.e., empagliflozin, dapagliflozin, canagliflozin, ertugliflozin) with placebo;

3. trials reporting prespecified desired CV, kidney and safety outcomes; and

4. follow-up duration lasted for at least 6 months. There were no limitations on the language or sample size of RCTs.

The main outcomes of interest were all-cause mortality, CV mortality, HHF, MI, stroke, and kidney outcome, which was defined as the composite of end-stage kidney disease, a doubling of the serum creatinine level, or kidney related mortality.

**Comparison assessed**

Figure 2a. All-Cause Mortality. SGLT vs placebo

**Results**

**18. Zou_2020**

**Title of the systematic review**

Sodium-glucose cotransporter inhibitors as add-on therapy in addition to insulin for type 1 diabetes mellitus: A meta-analysis of randomized controlled trials

**Inclusion criteria**

Population= T1DM.

1. a randomized controlled clinical trial assessing the efficacy and safety of SGLT inhibitors versus a placebo;

2. studies with data types providing categorization and continuity data;

3. articles written in English or Chinese; and

4. studies involving human subjects.

Outcomes: FBG, HbA1c, Mean amplitude of glucose excursion (MAGE), Daily total insulin dose (units per day), complications: Diabetic ketoacidosis (DKA), Urinary tract infections, genital infections.

**Comparison assessed**

Figure 4d. Urinary tract infections. SGLT inhibitors vs a placebo

**Results**

**19. Yates_2020**

**Title of the systematic review**

Venous thromboembolism risk with JAK inhibitors: A Meta-analysis

**Inclusion criteria**

Phase II and III RCTs of JAKi therapy with a placebo comparator arm.

Doses of JAKi therapies (tofacitinib 5mg and 10mg twice daily, Baricitinib 2mg and 4mg once daily Upadacitinib 15mg once daily) that were licensed when the literature search was performed (September 2020) were considered.

Filgotinib 200mg and 100mg was included having just received marketing authorisation from the European Commission for the treatment of rheumatoid arthritis.

Serious adverse events (SAEs) and adverse events (AEs). Deep vein thrombosis (DVT) and pulmonary embolus (PE) were considered VTE events.

**Comparison assessed**

Figure 2. Forest plot of overall VTE events. JAK inhibitors vs placebo

**Results**

**20. Dave_2020**

**Title of the systematic review**

Comparative Effectiveness of Entecavir Versus Tenofovir for Preventing Hepatocellular Carcinoma in Patients with Chronic Hepatitis B: A Systematic Review and Meta-Analysis

**Inclusion criteria**

(RCTs) and observational studies that met the following inclusion criteria:

1. patients, adults (age >18 years) with chronic HBV

infection (generally defined as hepatitis B surface antigen persisting for at least 6 months);

2. Intervention, ETV;

3. control, TDF;

4. outcome, risk of HCC.

Due to a paucity of randomized trials addressing the comparative efficacy of the two antiviral agents for reducing risk of HCC (related to short follow-up duration, small sample size, and low event rate), we a priori opted to include observational studies in this meta-analysis.

**Comparison assessed**

Figure 2. Forest plot of incidence of HCC in patients with CHB treated with ETV and TDF, unadjusted analysis

**Results**
